# Additive-Free Contact-Electro-Catalysis/Vacuum Ultraviolet System for Rapid Mitigation of Antimicrobial-Resistance-Associated Contaminants in Water

**DOI:** 10.34133/research.1348

**Published:** 2026-07-01

**Authors:** Weixin Li, Xinyu Xing, Kai Yang, Jikai Sun, Jialuo Tu, Zihan Liang, Fang Cao, Shanshan Wang, Jianing Dong, Li Cui, Yong-Guan Zhu, Zhong-Qun Tian, Feng Ru Fan

**Affiliations:** ^1^ State Key Laboratory of Physical Chemistry of Solid Surfaces, iChEM, College of Chemistry and Chemical Engineering, Innovation Laboratory for Sciences and Technologies of Energy Materials of Fujian Province (IKKEM), Xiamen University, Xiamen 361005, China.; ^2^ State Key Laboratory of Regional and Urban Ecology, Institute of Urban Environment, Chinese Academy of Sciences, Xiamen 361021, China.; ^3^ University of Chinese Academy of Sciences, Beijing 100085, China.; ^4^ Department of Chemical and Environmental Engineering, University of California, Riverside, Riverside, CA 92521, USA.

## Abstract

The widespread occurrence of antimicrobial-resistance-associated contaminants in natural and engineered water systems poses a major threat to environmental and public health. Conventional contact-electro-catalysis (CEC) can generate interfacial H_2_O_2_ and reactive oxygen species, but its efficiency is often constrained by sluggish H_2_O_2_ activation and limited reactive oxygen species flux. Here, we report an additive-free contact-electro-catalysis/vacuum ultraviolet (CEC–VUV) system that uses low-cost polytetrafluoroethylene as a dielectric catalyst for rapid mitigation of antibiotics, pathogens, and antibiotic resistance genes in water. Mechanistic analyses reveal that VUV irradiation activates CEC-derived H_2_O_2_ and O_3_ through charge–photon coupling, converting these otherwise underutilized intermediates into ^•^OH. CEC continuously supplies interfacial charges and H_2_O_2_, while VUV promotes H_2_O_2_ photolysis and O_2_-to-O_3_ conversion. The generated O_3_ is further activated at the charged polytetrafluoroethylene/water interface, establishing an interconnected H_2_O_2_/O_3_-to-^•^OH conversion network that amplifies ^•^OH generation. This mechanism enables complete degradation of representative antibiotics within 10 min, complete inactivation of pathogenic bacteria within 5 min, effective antibiotic resistance gene removal in real water matrices, and stable bactericidal performance under continuous-flow operation.

## Introduction

**Fig. 1.**
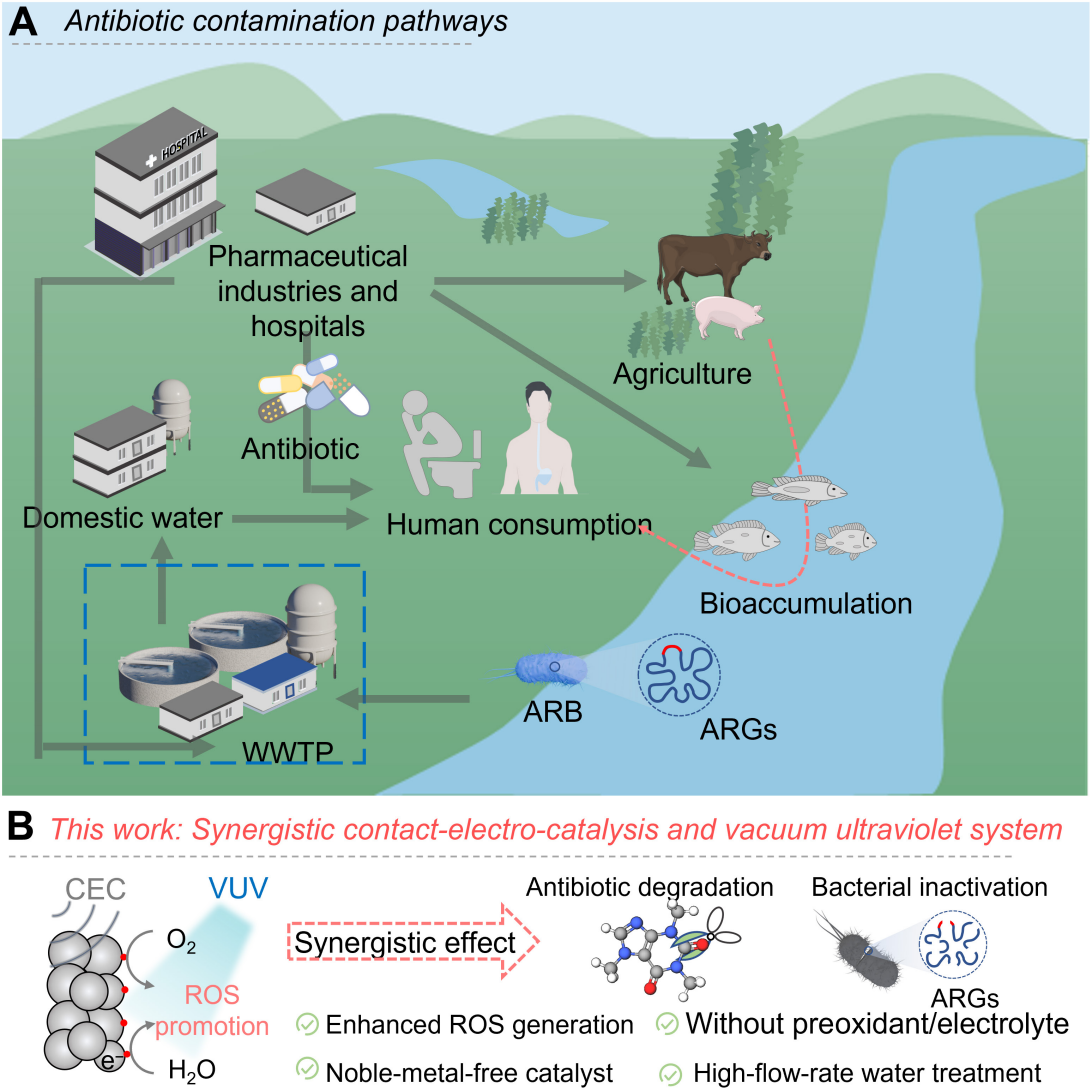
Overview of the spread of antibiotic resistance in the environment and methods for elimination. (A) Conventional water treatment technologies and emerging photocatalytic and electrocatalytic water treatment technologies. (B) Contact-electro-catalysis/vacuum ultraviolet (CEC–VUV) synergistic water treatment system.

The widespread presence of antibiotic residues, antibiotic-resistant bacteria, and antibiotic resistance genes (ARGs) in water systems represents a critical and escalating threat to global health and environmental safety. While antibiotics are essential for infection control, their extensive use and improper disposal result in persistent accumulation in both natural and engineered water systems (Fig. 1A). These residual antibiotics in turn impose strong selective pressure to promote the emergence, proliferation, and horizontal transfer of ARGs even to clinical pathogens, undermining both public health and ecosystem stability [1–4]. Green, efficient water disinfection technologies that eliminate antibiotics, pathogens, and ARGs are critical for protecting human health, ensuring ecological safety, and advancing United Nations Sustainable Development Goals. Traditional disinfection methods such as chlorination or ultraviolet (UV) treatment have significant limitations when dealing with complex pollution systems containing antibiotics and ARGs. Chlorination generates carcinogenic disinfection by-products through reactions with organic matter, requiring continuous chemical inputs [5]. UV disinfection, although residue-free, is hindered by water turbidity and often fails to fully inactivate ARGs. These limitations highlight the need for sustainable, energy-efficient technologies that target ARGs with minimal secondary risks.

Hydrogen peroxide (H_2_O_2_), a broad-spectrum, residue-free oxidant, holds great promise for water treatment and green chemical synthesis. Compared to H_2_O_2_ itself, the hydroxyl radicals (^•^OH) produced during its decomposition possess a much higher oxidation potential and are the true reactive species. Advanced oxidation processes (AOPs), such as H_2_O_2_ activation by iron ions or persulfate activation to generate sulfate radicals, offer similar oxidative power. However, they require presynthesized radical precursors and stabilizers to prevent self-decomposition, reducing efficiency and practicality. Photocatalytic and electrocatalytic systems generate reactive oxygen species (ROS) in situ, avoiding the need for external reagents [6,7]. Despite their strong oxidative potential, photocatalysis is hindered by poor light penetration in water, while electrocatalysis relies on costly noble metal catalysts, limiting economic viability. Both processes often require extended treatment times, which substantially reduce overall efficiency [8,9]. An ideal water disinfection technology should be safe, efficient, affordable, durable, and adaptable for decentralized use in resource-limited regions. Developing a technology that meets all these criteria simultaneously remains a marked challenge. Integrated photocatalysis, electrocatalysis, and ultrasonic degradation with AOPs generate highly reactive species to degrade persistent pollutants into CO_2_ and H_2_O, offering innovative solutions for sustainable water treatment [10–14]. Many studies have explored the synergistic generation of ^•^OH through combinations of ultrasound, O_3_, H_2_O_2_, and (V)UV, revealing that ^•^OH plays a highly reactive and crucial role in the degradation of antibiotics [15–18]. However, these systems often face rapid H_2_O_2_ decomposition under ultrasound or UV irradiation, resulting in inefficient radical utilization [19–21]. Moreover, while O_3_ is low-cost, its conversion efficiency to ^•^OH is limited.

Contact-electro-catalysis (CEC), an emerging catalytic approach, offers distinct advantages including low cost, simple operation, and strong durability. Based on the ubiquitous phenomenon of contact electrification at 3-phase interfaces, CEC drives chemical reactions through electron transfer [22,23]. Using commercial fluorinated polymers as catalysts, water and oxygen can be transformed into ROS under ultrasonic activation [24–26]. Owing to their excellent dielectric properties, fluorinated polymers tend to attract electrons from water molecules upon contact, inducing electron transfer and creating a strong interfacial electric field. This field lowers reaction energy barriers and facilitates ROS formation [27,28]. However, compared with photocatalytic and electrocatalytic methods, CEC alone produces relatively limited amounts of ROS. To address this limitation, strategies such as introducing Fe ions to activate in situ H_2_O_2_ or utilizing organic solvents like acetonitrile to accelerate radical generation have been proposed [29,30]. Although effective, these approaches carry the risk of secondary pollution and increased maintenance costs due to the continuous addition of chemical agents.

**Fig. 2.**
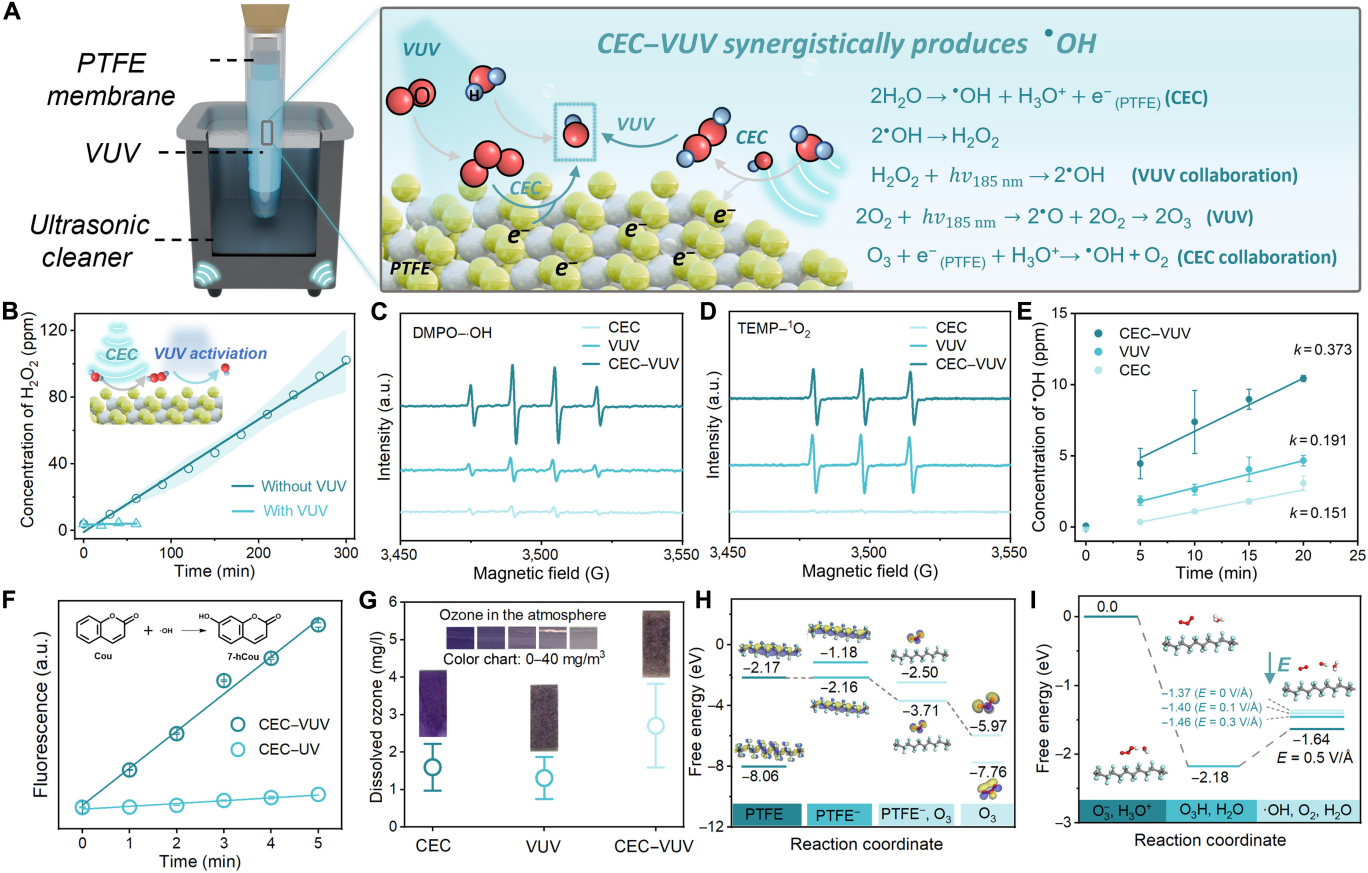
Reactive oxygen species (ROS) generation mechanism and efficiency in the contact-electro-catalysis/vacuum ultraviolet (CEC–VUV) system. (A) Schematic illustration of the CEC–VUV synergistic system. (B) H_2_O_2_ concentrations generated by CEC with and without VUV irradiation. Electron paramagnetic resonance (EPR) signal intensities of (C) ^•^OH and (D) ^1^O_2_ detected in different systems. (E) Quantification of ^•^OH concentration using sodium benzoate as a probe. (F) Detection of ^•^OH concentrations in CEC–VUV and CEC–ultraviolet (UV) systems using coumarin. (G) O_3_ concentrations in gas and liquid phases under different systems. (H) Free energy change for electron transfer from the polytetrafluoroethylene (PTFE) surface to O_3_. (I) Energy required for the conversion of O_3_ to ^•^OH under different interfacial electric field strengths.

Here, we establish a synergistic contact-electro-catalysis/vacuum ultraviolet (CEC–VUV) platform that amplifies ROS generation and enables disinfection without external ion additives, using low-cost and chemically robust polytetrafluoroethylene (PTFE) as the catalyst (Fig. 1B and Fig. S1). Vacuum ultraviolet (VUV) is particularly well suited to pair with CEC because it complements electrification-driven chemistry: CEC continuously builds up interfacial charges and produces H_2_O_2_ in situ, whereas VUV efficiently converts these intermediates into highly reactive radicals and opens additional oxidation pathways. This charge–photon complementarity makes the coupling mechanistically coherent rather than a simple combination of 2 processes. In this system, PTFE-mediated CEC reactions generate H_2_O_2_ and a small amount of ^•^OH. Meanwhile, VUV light enhances the radical chain reaction through dual pathways: photons and hydrated electrons (e_aq_^−^) decompose H_2_O_2_ into ^•^OH, and high-energy UV excites O_2_ to generate O_3_, which is subsequently converted to ^•^OH under the strong interfacial electric field. Together, these processes construct an efficient oxidation system capable of in situ and sustained ^•^OH generation without the addition of external strong oxidants. Simultaneously, ultrasonic cavitation disrupts bacterial biofilms, improving radical penetration efficiency (Fig. 2A). Under this synergistic system, the steady-state ^•^OH concentration is substantially increased compared with those of the individual CEC and VUV systems, supporting rapid antibiotic degradation and pathogen inactivation, enabling complete antibiotic degradation within 10 min and full pathogenic bacterial inactivation within 5 min. The system maintains high bactericidal efficiency at a flow rate of 1,150 ml min^−1^ and shows effective removal of antibiotics, pathogens, and ARGs in real water matrices, indicating its potential for continuous-flow treatment. The CEC–VUV system not only ensures simple operation and minimal health risks but also is highly adaptable for decentralized applications, especially in small-scale or remote water treatment scenarios. These results suggest that the CEC–VUV system may serve as a promising additive-free strategy for ROS-driven water purification, particularly in decentralized treatment scenarios.

## Results

### Efficient ^•^OH generation performance of the CEC–VUV synergistic system

Utilizing the contact electrification effect, ultrasound provides mechanical energy to induce dipole polarization in PTFE molecules through deformation. When polarized PTFE contacts water, it captures electrons from water molecules, promoting water dissociation and ^•^OH formation. Owing to PTFE’s superior dielectric properties, these electrons are retained on its surface. However, the short lifespan of ^•^OH typically leads to rapid recombination into stable H_2_O_2_. In the CEC system, approximately 100 ppm H_2_O_2_ is generated within 5 h (Fig. S2). To convert this stable H_2_O_2_ into the more reactive ^•^OH, we integrated VUV light into the system, resulting in the H_2_O_2_ concentration dropping below the detection limit (Fig. 2B). Electron paramagnetic resonance (EPR) spectra confirmed ^•^OH generation in all 3 systems (CEC, VUV, and CEC–VUV). In the VUV system, multiple pathways contribute to ROS formation: homolysis and ionization of water molecules under 185-nm irradiation produce ^•^OH, hydrogen atoms (H^•^), and solvent (hydrated) electrons (e_aq_^−^) (Eqs. (1) and (2)). VUV photolysis of O_2_ produces O atoms that subsequently form O_3_ (Eq. (3)), and H_2_O_2_ photolysis yields ^•^OH (Eqs. (4) and (5) and Fig. S3) [31,32].

Compared to VUV irradiation alone, the CEC–VUV system markedly enhanced the ^•^OH radical concentration (Fig. 2C and Fig. S4). Notably, only the VUV and CEC–VUV systems generated substantial amounts of singlet oxygen (^1^O_2_) (Fig. 2D and Fig. S5). Quantitative analysis using sodium benzoate as an ^•^OH scavenger revealed that the CEC–VUV system achieved an ^•^OH generation rate of 0.373 ppm/min, surpassing the VUV and CEC systems by factors of 1.96 and 2.48, respectively (Fig. 2E). To distinguish the effect of ultrasound-assisted O_3_ dispersion from that of CEC, a control experiment (US–VUV, without PTFE) was performed. After 20 min, the ^•^OH concentration in the US–VUV system was 14.5 ppm lower than that in the CEC–VUV system, indicating that ultrasound alone can promote the utilization of VUV-generated O_3_, whereas CEC further enhances O_3_ activation and its conversion into ^•^OH (Figs. S6 and S7).

Using coumarin as an ^•^OH probe, the formation of 7-hydroxycoumarin under 350-nm excitation exhibited a characteristic fluorescence peak at 450 nm, corroborating the higher ^•^OH production in the CEC–VUV system, consistent with sodium benzoate and EPR results (Fig. S8). Further experiments exploring the effects of 185- and 254-nm light wavelengths revealed that the ^•^OH concentrations in the CEC–VUV system were significantly higher than those in the CEC–UV system. As 254-nm light is less effective at generating O_3_, these findings underscore the critical role of O_3_ in ^•^OH production (Fig. 2F and Fig. S9). Quantitative analysis of O_3_ concentrations showed that both the CEC and VUV systems produced dissolved O_3_, with the CEC–VUV system yielding even higher levels. Upon 185-nm irradiation, O_2_ in the gas phase efficiently converts into O_3_, enhancing ROS availability (Fig. 2G, Fig. S10, and Table S1).

Building on previous reports, O_3_ can accept electrons to form O_3_^−^, which then reacts with H_3_O^+^ to form HO_3_^•^, subsequently decomposing into ^•^OH and O_2_ upon gaining another electron [33,34]. We propose that during CEC, PTFE acts as an electron carrier, capturing electrons from water and transferring them to O_3_. Mulliken charge distribution analysis shows that after electron transfer, O_3_ carries a charge of −0.991 e, while PTFE retains only −0.009 e, indicating nearly complete electron transfer (Eq. (6) and Fig. S11). The generated O_3_^−^ then reacts with H_3_O^+^ to form HO_3_^•^ and H_2_O (Eq. (7)), and O_3_H decomposes into ^•^OH and O_2_ (Eq. (8)). Molecular orbital analysis revealed that electrons transfer spontaneously from the highest occupied molecular orbital (HOMO) of PTFE (−2.16 eV) to the lowest unoccupied molecular orbital (LUMO) of O_3_ (−5.97 eV), forming a new HOMO at −3.71 eV in the PTFE–O_3_^−^ complex (Fig. 2H). Due to its electret-like properties, PTFE gradually accumulates charge during the CEC process, creating a strong interfacial electric field. Free energy calculations show that the reduction of HO_3_^•^ to ^•^OH is markedly favored under an electric field: at 0.5 V/Å, the free energy drops to −1.64 eV, compared to −1.37 eV at 0 V/Å (Fig. 2I), demonstrating that the interfacial electric field critically promotes ROS formation.

$$H_{2}O+\mathrm{h\nu}\;\left(185\;\mathrm{nm}\right)\to^{•}\mathrm{OH}+H^{•}$$ (1)

$$H_{2}O+\mathrm{h\nu}\;\left(185\;\mathrm{nm}\right)\to^{•}\mathrm{OH}+H^{+}+e^{-}\left(\mathrm{aq}\right)$$ (2)

$$3O_{2}+\mathrm{h\nu}\;\left(185\;\mathrm{nm}\right)\to {\text{2O}}_{3}$$ (3)

$$H_{2}O_{2}+\mathrm{h\nu}\;\left(185/254\;\mathrm{nm}\right)\to 2^{•}\mathrm{OH}$$ (4)

$$H_{2}O_{2}+e^{-}\left(\mathrm{aq}\right)\to^{•}\mathrm{OH}+{\mathrm{OH}}^{-}$$ (5)

$$O_{3}+e^{-}\left(\text{PTFE}\right)\to {O_{3}}^{•-}$$ (6)

$${O_{3}}^{•-}+H_{3}O^{+}⇌{{\mathrm{HO}}_{3}}^{•}+H_{2}O$$ (7)

$${{\mathrm{HO}}_{3}}^{•}\to^{•}\mathrm{OH}+O_{2}$$ (8)

### Antibiotic degradation by the CEC–VUV synergistic system

**Fig. 3.**
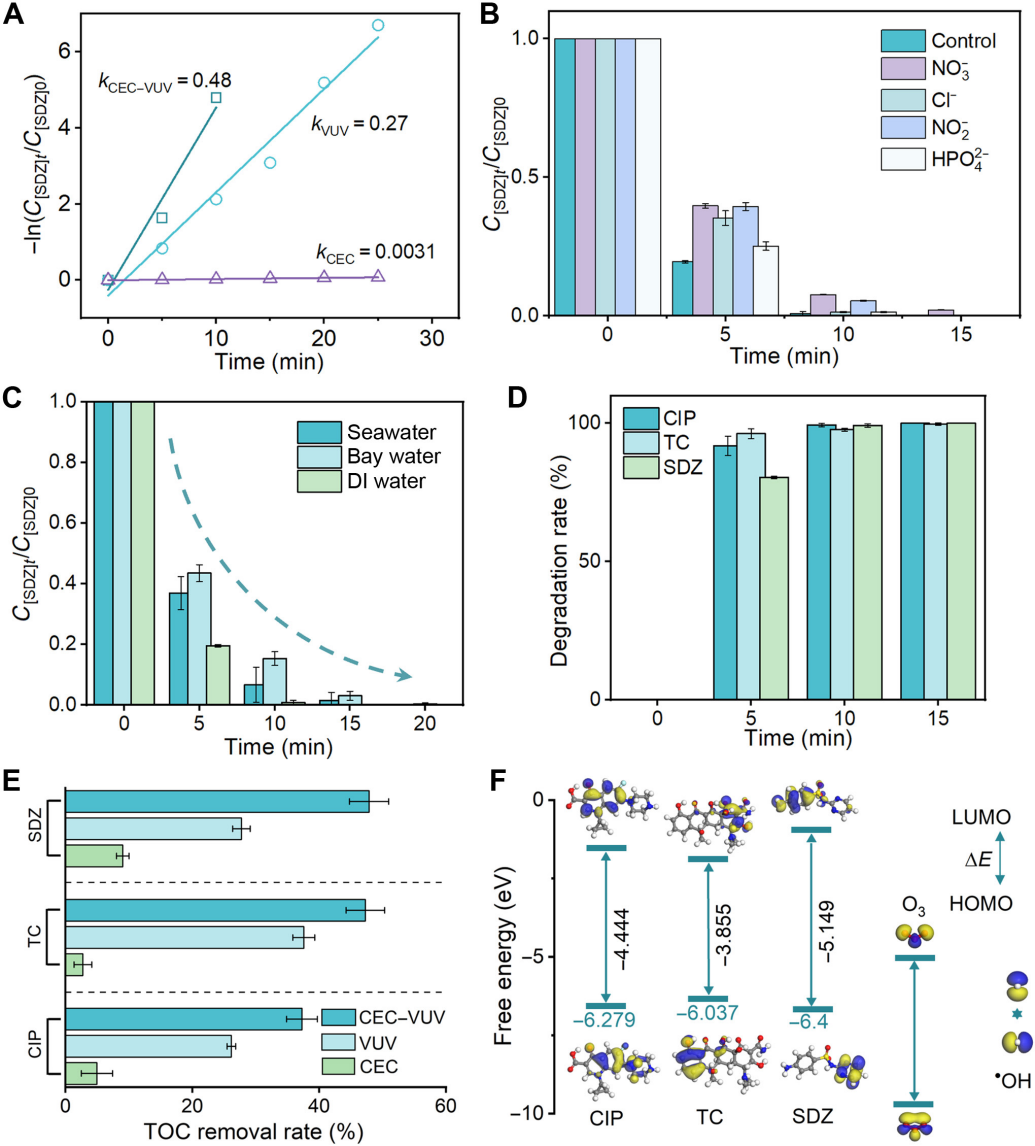
Degradation performance of antibiotics by the synergistic contact-electro-catalysis/vacuum ultraviolet (CEC–VUV) system. (A) Comparison of kinetic rate constants for sulfadiazine (SDZ) degradation in different systems. (B) SDZ degradation performance of the CEC–VUV system in the presence of various environmental ions. (C) SDZ degradation efficiency in seawater, bay water, and deionized water using the CEC–VUV system. (D) Versatility of the CEC system in degrading different types of antibiotics (ciprofloxacin [CIP] and tetracycline hydrochloride [TC]). (E) Total organic carbon (TOC) removal efficiencies of CIP, TC, and SDZ in different systems. (F) Free energy levels of antibiotics (CIP, TC, and SDZ) and reactive species (O_3_ and ^•^OH).

Sulfadiazine (SDZ), a widely used sulfonamide antibiotic characterized by a stable aniline-heterocycle sulfonamide structure, serves as a model contaminant due to its chemical resilience. In 25-min degradation experiments, the kinetics of SDZ degradation in CEC, VUV, and CEC–VUV systems all followed a pseudo-first-order model (Fig. 3A). The CEC system, limited by low ^•^OH generation, exhibited a rate constant *k* of 0.0031 min^−1^. The VUV system achieved better performance due to O_3_ formation and photolysis. Notably, in the synergistic CEC–VUV system, the chain reaction between ^•^OH and O_3_ was notably enhanced, leading to complete degradation of SDZ within 10 min, reaching a rate constant of 0.48 min^−1^, a 77% improvement over that of the VUV system alone, confirming the synergistic enhancement mechanism of the CEC–VUV system. Compared to the CEC–VUV system, the VUV/H_2_O_2_ system exhibits a faster degradation rate of SDZ within the initial 2 min, followed by a significant slowdown.

To elucidate SDZ degradation pathways, high-performance liquid chromatography–mass spectrometry (HPLC–MS) was employed to identify intermediates formed during ^•^OH-mediated reactions (Fig. S12). The peak at a 1.8-min retention time corresponded to SDZ, while VUV excitation triggered SO_2_ elimination, yielding a degradation product (*m*/*z* 175.1). Based on the analysis of major peaks, a possible ^•^OH-mediated degradation pathway was proposed (Fig. S13) [35–37]. As the reaction proceeded, although P6 still showed some toxicity, the toxicity of P4 and P5 was significantly lower than that of P1, P2, and the original SDZ, indicating that with the progress of the reaction, SDZ was gradually degraded into low-toxicity small molecules and underwent further oxidation and partial mineralization, as supported by total organic carbon removal (Fig. S14). To further evaluate the toxicity changes during SDZ degradation, a bioluminescence inhibition assay using *Aliivibrio fischeri* was performed. The treated solution showed no detectable increase in acute toxicity under the tested conditions (Fig. S15, one-way analysis of variance, *P* = 0.132).

In natural waters, inorganic anions, natural organic matter, and suspended particles may suppress ^•^OH-driven degradation by scavenging radicals or attenuating VUV irradiation. We therefore examined SDZ degradation in the presence of representative anions, real water samples, and model interferents. At 10 mM, HPO_4_^2−^ showed the weakest inhibition because of its lower reactivity toward ^•^OH, whereas NO_3_^−^, NO_2_^−^, and Cl^−^ caused stronger inhibition to varying degrees [38–42]. Nevertheless, complete SDZ degradation was achieved within 15 min in all anion-containing systems (Fig. 3B). In urban water and seawater, SDZ was fully degraded within 20 min (Fig. 3C). To further impose organic-matter and turbidity stress, 20 ppm humic acid and 100 ppm kaolin were introduced as model interferents. The degradation remained rapid, with *k* decreasing only from 0.63 to 0.61 min^−1^ with humic acid and 0.56 min^−1^ with kaolin while still achieving >99% removal within 10 min (Fig. S16). These results demonstrate that the CEC–VUV system maintains strong SDZ degradation performance despite radical competition and light attenuation.

Beyond SDZ, the CEC–VUV system effectively degraded other types of antibiotics, including tetracyclines (e.g., tetracycline hydrochloride [TC]) and fluoroquinolones (e.g., ciprofloxacin [CIP]). These antibiotics are extensively detected in various environments (Fig. 3D and Fig. S17). In this system, synergistically generated ^•^OH, as the most potent oxidizing species, rapidly oxidizes antibiotics, while O_3_ produced by VUV exhibits a high oxidative capacity despite a slower rate. Physical effects, such as light irradiation and ultrasound, simultaneously enhance the interaction between ROS and antibiotics. This physicochemical synergy leads to competitive degradation performance compared with selected reported ultrasound- and ultrasound/light-assisted systems under similar conditions (Tables S2 and S3). After 20 min of reaction, the synergistic system achieved higher total organic carbon removal rates for all 3 antibiotics than VUV and CEC (Fig. 3E).

A smaller HOMO–LUMO energy gap indicates higher reactivity, while a larger gap reflects greater stability. With a bandgap of zero, ^•^OH exhibits notably higher reactivity than O_3_ (Fig. 3F). In the ^•^OH-mediated radical pathway, the strong electron-withdrawing capacity of ^•^OH enables efficient electron extraction from antibiotic molecules. A higher HOMO level indicates greater susceptibility to electron loss, while a lower LUMO level facilitates electron gain. SDZ’s lower HOMO level suggests greater resistance to ^•^OH oxidation compared to TC and CIP, consistent with experimental findings (TC > CIP > SDZ).

### Pathogen inactivation performance of the CEC–VUV synergistic system in water

Waterborne pathogens, particularly those with antimicrobial resistance (AMR), in contaminated drinking water drive morbidity and mortality, especially in resource-limited rural communities, by causing treatment failures and spreading ARGs [43,44]. Therefore, a comprehensive evaluation should not only quantify the disinfection efficacy of the CEC–VUV system against pathogenic bacteria but also systematically assess its ability to prevent the horizontal transfer of ARGs within residual microbial communities.

**Fig. 4.**
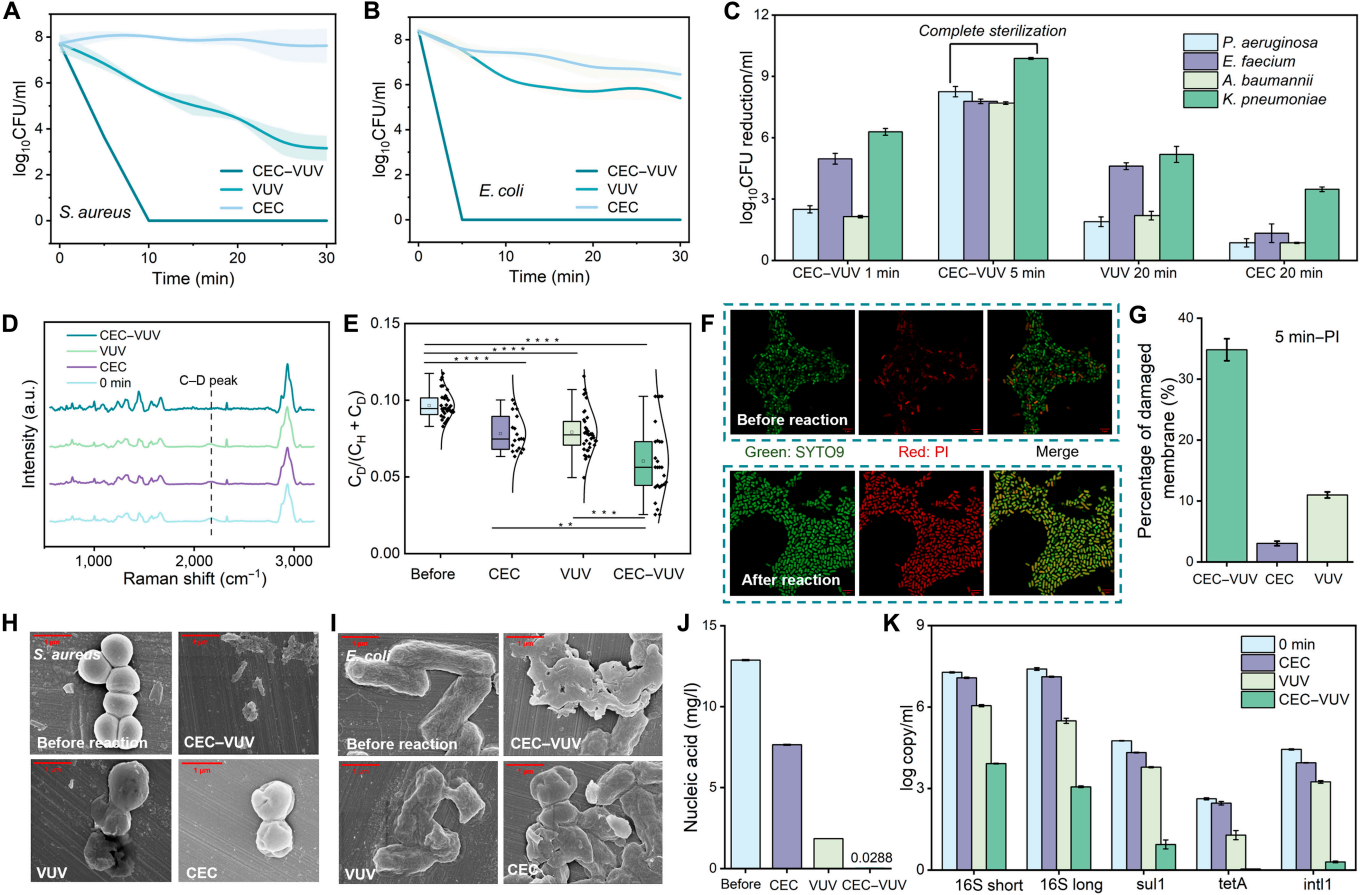
Contact electro-catalysis/vacuum ultraviolet (CEC–VUV) for water disinfection. The inactivation efficiency of ESKAPE pathogens (A) *Staphylococcus aureus*, (B) *Escherichia coli*, and (C) *Pseudomonas aeruginosa*, *Enterococcus faecium*, *Acinetobacter baumannii*, and *Klebsiella pneumoniae* with different systems. (D) Average single-cell Raman spectroscopy of bacteria (before and after different systems) labeled with deuterium oxide (D_2_O) to indicate their metabolic state. (E) Distribution quartiles of the C–D Raman ratios of *E. coli* indicative of metabolic activities measured from around 25 randomly selected single cells before and after different systems. Statistical significance was determined by the Kruskal–Wallis *H* test followed by Dunn’s multiple comparisons test. Pairwise comparisons showed that CEC–VUV was significantly higher than both CEC and VUV, and CEC was significantly higher than VUV (2 asterisks [**], 3 asterisks [***], and 4 asterisks [****] represent statistically significant results at the 99%, 99.9%, and 99.99% confidence levels corresponding to *P* < 0.01, *P* < 0.001, and *P* < 0.0001, respectively). (F) Laser scanning confocal microscopy (LSCM) images of *E. coli* before and after CEC–VUV treatment. (G) Percentage of *E. coli* with damaged membranes after different systems measured as propidium iodide (PI) fluorescence intensity. Statistical significance was determined by one-way analysis of variance (ANOVA) followed by Tukey’s honestly significant difference (HSD) post hoc test. The overall group difference was highly significant (*F*(2,6) = 662.260, *P* = 9.17 × 10^−8^). Scanning electron microscopy (SEM) images of *S. aureus* (H) and *E. coli* (I) before and after different systems (CEC–VUV, VUV, and CEC). (J) Concentration of environmental DNA in Xinglin Bay water before and after different systems. Statistical significance was analyzed by one-way ANOVA followed by Tukey’s HSD post hoc test. A highly significant overall difference was observed among the before, CEC–VUV, VUV, and CEC groups (*F*(3, 8) = 122,078.45, *P* < 0.0001), and all pairwise comparisons were highly significant (*P* < 0.0001). (K) Relative abundance of 1,465- and 60-bp 16S ribosomal RNA (rRNA) fragments, antibiotic resistance genes (sul1 and tetA), and integron gene (intl1) in Xinglin Bay water samples before and after different systems.

The ESKAPE pathogens (*Enterococcus faecium*, *Staphylococcus aureus*, *Klebsiella pneumoniae*, *Acinetobacter baumannii*, *Pseudomonas aeruginosa*, and *Enterobacter* species) are prevalent in clinical settings and have been designated as priority pathogens that require urgent attention by the World Health Organization (WHO) [44,45]. To assess the disinfection efficacy of the CEC–VUV synergistic system, sterilization experiments were conducted on all 6 ESKAPE bacteria. At an initial concentration of 10^8^ colony-forming units (CFU)/ml, the gram-positive and gram-negative model pathogens (*S. aureus* and *Escherichia coli*) were completely inactivated (100%) within 10 and 5 min, respectively, as determined by the plate-counting method. By comparison, the CEC system exhibited negligible or slight inactivation, and the VUV system achieved only partial disinfection even after 30 min (Fig. 4A and B and Figs. S18 and S19). The inactivation efficiency, under the same treatment duration, was significantly greater than that of ozone-based disinfection (Fig. S20). The CEC–VUV also works well in the other 4 ESKAPE strains by generating abundant ROS, achieving complete inactivation within 5 min. Notably, the treatment of the CEC–VUV system within just 1 min already outperformed 20-min VUV or CEC treatments within just 1 min (Fig. 4C).

It is worth noting that traditional culturing methods assess only the ability of bacteria to form colonies after disinfection, potentially underestimating their viability. Many waterborne pathogens in drinking water, including *Helicobacter pylori* and *P. aeruginosa*, have been reported to enter a viable but nonculturable (VBNC) state after water treatment such as chlorination (NaClO), UV radiation, and peracetic acid [46,47]. These VBNC bacteria can persist and potentially resuscitate under favorable conditions such as nutrient availability and temperature changes, posing notable risks to water safety and potential transfer of ARGs [48,49]. To investigate the efficacy of the CEC–VUV system against VBNC bacteria, we employed single-cell Raman spectroscopy coupled with deuterium oxide (D_2_O) labeling to assess bacterial metabolic activity [50]. This method quantifies metabolic activity based on the Raman shift from C–H to C–D bands upon deuterium incorporation, enabling single-cell-level resolution of heterogeneous activity [51]. After 5-min treatment with the CEC, VUV, and CEC–VUV systems, *E. coli* was then incubated in Luria–Bertani (LB) medium containing 50% D_2_O for 12 h. The C_D_/(C_D_ + C_H_) ratio from single-cell Raman spectra, serving as an indicator of bacterial metabolic activity, was used to evaluate whether the CEC–VUV system can effectively address the limitation of conventional disinfection methods in eliminating residual VBNC bacteria. Compared to other treatments, the CEC–VUV group showed a one-third reduction in the C_D_/(C_D_ + C_H_) ratio, indicating considerably lower bacterial metabolic activity and effective suppression of VBNC cells (Fig. 4D and E). This is crucial for minimizing health risks associated with VBNC bacteria.

To elucidate the inactivation mechanism, live/dead fluorescence assays using propidium iodide (PI) and SYTO9 were used to evaluate the impact of CEC–VUV treatment on bacterial cell membrane integrity [52]. SYTO9, which emits green fluorescence, can enter all bacterial cells and is used to assess total cell counts, whereas PI, which emits red fluorescence, penetrates only cells with damaged membranes. *E. coli* was stained with SYTO9 and PI after 5 min of treatment. Laser scanning confocal microscope images revealed that, in contrast to the minimal cell death observed before treatment, CEC–VUV treatment caused extensive bacterial damage, with the vast majority of cells emitting red fluorescence (Fig. 4F). Flow cytometry was subsequently used to quantify fluorescence intensity and assess the live/dead cell ratio. The CEC–VUV group showed a significantly higher red fluorescence intensity than the CEC and VUV groups, indicating substantial membrane disruption that allowed PI to penetrate compromised cells (Fig. 4G). Furthermore, scanning electron microscopy (SEM) revealed severe morphological disruption and cell deformation, fragmenting bacterial cells into debris (Fig. 4H and I and Fig. S21).

Considering that intracellular DNA including ARGs may be released following bacterial lysis, increasing AMR spread risk via transformation, we further assessed the ability of CEC–VUV to degrade DNA and ARGs. Natural water samples from Xinglin Bay (in Xiamen) were analyzed for DNA content before and after disinfection. The CEC–VUV system achieved a DNA removal rate of 99.78%, markedly higher than those achieved by CEC (40.62%) and VUV (85.62%) (Fig. 4J). Gel electrophoresis confirmed the no visible DNA bands were observed after CEC–VUV treatment, indicating substantial DNA degradation below the detection limit of gel electrophoresis (Fig. S22).

Quantitative polymerase chain reaction analysis confirmed that the CEC–VUV system effectively reduced not only ARGs (sul1 and tetA) but also mobile genetic elements, as evidenced by the significant decrease in the abundance of the integron gene (intl1). In addition, both long-chain (1,400-bp) and short-chain (40-bp) DNA fragments were substantially degraded, suggesting broad-spectrum efficacy against various ARGs and mobile genetic elements. These results indicate that the system not only removes antibiotic-resistant bacteria but also mitigates the risk of their further dissemination by targeting residual genetic materials (Fig. 4K and Table S4). These findings confirm that the CEC–VUV system induces extensive damage to bacterial membranes and cellular structures, achieving complete pathogen inactivation. Moreover, the suppression of VBNC cells, along with the removal of residual genetic material, may reduce the potential risk associated with horizontal ARG transfer, highlighting its potential for advanced water disinfection applications.

### Catalyst stability and continuous-flow reaction performance

**Fig. 5.**
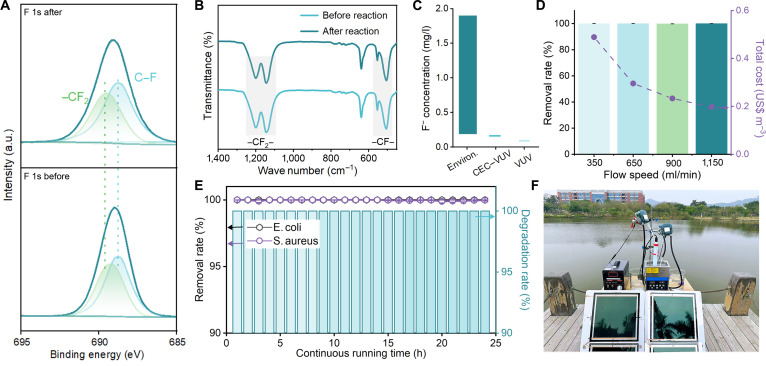
Catalyst stability and performance of the continuous-flow system. (A) F 1s x-ray photoelectron spectroscopy (XPS) spectra of the PTFE membrane before and after the reaction. (B) Fourier transform infrared (FTIR) spectra of the polytetrafluoroethylene (PTFE) membrane before and after the reaction. (C) Comparison of F^−^ concentrations in the contact-electro-catalysis/vacuum ultraviolet (CEC–VUV) and VUV systems after 30 min of reaction with environmental F^−^ background levels. (D) Bacterial removal efficiency of *Staphylococcus aureus* by CEC–VUV under different flow rates, along with the corresponding electricity cost and total operating cost per cubic meter. (E) Disinfection performance against *Escherichia coli* and *S. aureus* (>10^6^ colony-forming units [CFU]/ml) and degradation efficiency of 0.1 mg/l sulfadiazine (SDZ) at a flow rate of 650 ml/min. (F) Schematic diagram of the solar-powered CEC–VUV system.

Despite the intense oxidative environment created by VUV irradiation, PTFE showed good stability under the tested CEC–VUV conditions due to its inherent chemical inertness. After 10 reaction cycles for antibiotic degradation, the surface of the PTFE membrane is affected by ultrasound and wrinkles appear, which is caused by the high-frequency vibration of ultrasound (Fig. S23). X-ray photoelectron spectroscopy characterization of the PTFE membrane surface revealed a slight increase in the binding energy of the –CF_2_ functional group (Δ*E* = 0.3 eV), while the C–F bond energy showed no notable change (Fig. 5A). This shift indicates mild defluorination, with residual –CF*_x_* (*x* < 2) groups reducing the electron density around remaining fluorine atoms (Fig. S24), which is consistent with the slight decrease in the static water contact angle after cycling (Fig. S25). However, Fourier transform infrared spectroscopy detected no marked changes in the spectra, indicating that defluorination is minimal and confined to the interfacial region, with the bulk material remaining largely unaffected (Fig. 5B). To evaluate the environmental risk of defluorination by-products, ion chromatography was used to measure fluoride ion concentrations in the solution during the first 30 min of reaction (sufficient for complete antibiotic degradation within 15 min). The fluoride concentration was only 0.171 mg/l (Fig. 5C and Fig. S26), well below the WHO guideline for drinking water (0.5 to 0.7 mg/l) and typical background levels in natural water bodies (0.19 to 1.9 mg/l, equivalent to 10 to 100 μM) [53]. These findings suggest that fluoride release from interfacial defluorination remained limited under the tested conditions.

Leveraging these mechanisms, we developed a continuous-flow reactor (Fig. S27), achieving dynamic water balance by precisely controlling the inlet and outlet flow rates. Sterilization experiments targeting high concentrations of pathogenic *S. aureus* (>10^6^ CFU/ml) achieved >99.999% disinfection efficiency across various flow rates. At an optimized flow rate of 1.15 l/min, combined with the low cost of PTFE membranes, the total operational cost was maintained at US$0.197/m^3^ (Fig. 5D). During 24-h continuous operation (650 ml/min for 24 continuous hours), the system sustained >99.9% removal efficiency for both *E. coli* and *S. aureus*, with the residual SDZ concentration in the effluent below the detection limit (LOD = 0.01 μg/l) (Fig. 5E). The 48-h-treated PTFE membrane was further rinsed and reused for SDZ degradation to evaluate its retained catalytic activity. Although the postcycled PTFE showed a slightly slower initial degradation rate than fresh PTFE, it still achieved nearly complete SDZ removal within 10 min, indicating that its catalytic performance was largely preserved after prolonged operation in the simulated complex water matrix (Figs. S28 and S29). Based on these results, we assembled a prototype CEC–VUV device (Fig. 5F). This device utilizes advanced perovskite solar panels as a sustainable power source for the ultrasonic and VUV components [54]. Each solar panel delivers approximately 15 Wh, and connecting 8 panels in series meets the power requirements for a distributed water treatment system. This design highlights the system’s suitability for decentralized applications, particularly in small-scale or remote areas with limited infrastructure.

## Discussion

This study presents a CEC–VUV system for additive-free water disinfection and mitigation of AMR-associated contaminants, including antibiotics, pathogens, and ARGs. Utilizing cost-effective and durable PTFE as the catalyst, the system enables in situ generation of ROS, particularly ^•^OH, a highly reactive oxidizing species, which rapidly oxidizes antibiotics without the need for external chemical additives. This is achieved through a dual mechanism: H_2_O_2_ produced by CEC is decomposed into ^•^OH by VUV light, while O_3_ generated by VUV is activated by electrons transferred from the PTFE surface. The system completely degraded representative antibiotics (SDZ, TC, and CIP) within 10 min and achieved 99.9999% inactivation of all ESKAPE bacterial pathogens in 5 to 10 min. Moreover, the system effectively eliminated ARGs and markedly reduced VBNC bacteria by inducing severe membrane and intracellular damage, contributing substantially to the mitigation of AMR dissemination risk. This approach helps address the limited ROS utilization in conventional CEC/AOP systems, substantially enhancing both disinfection and degradation performance. Catalyst stability tests confirmed the minimal environmental impact from fluoride release, while continuous-flow experiments demonstrated robust long-term performance with low energy consumption (1.594 kWh/m^3^) and operational costs (US$0.197/m^3^). Overall, the CEC–VUV system offers substantial potential for practical water treatment, particularly in small-scale or remote settings.

## Materials and Methods

### Chemicals and materials

Sodium chloride (NaCl, ACS, ≥99%) was from Sinopharm, sodium sulfate (Na_2_SO_4_, ACS, ≥99%) was from Sinopharm, sodium nitrate (NaNO_3_, ACS, ≥99%) was from Sinopharm, sodium dihydrogen phosphate (NaH_2_PO_4_, AR, 99%) was from Sinopharm, tertiary butanol (AR, ≥99.5%) was from Sinopharm, furfuryl alcohol was from Aladdin (AR, ≥99%), methanol (AR, ≥99%) was from Sinopharm, 5,5-dimethyl-1-pyrroline *N*-oxide (97%) was from Energy Chemical, and 2,2,6,6-tetramethyl-4-piperidone hydrochloride was from Aladdin. Benzoic acid (ACS, 99.5%) was from Aladdin, titanium potassium oxalate (ACS, 98%) was from Aladdin, SDZ was from Macklin, CIP was from Macklin, TC was from Aladdin, lysogeny broth was from HOPEBIO, PI was from InvivoChem, 2× Taq Master Mix (Dye Plus) was from Vazyme, DL2000 Plus DNA Marker was from Vazyme, and D_2_O (99.0%) was from Sigma.

### Catalytic reactions

The reaction setup comprises a glass tube (20 cm long, 3 cm diameter) housing a commercial PTFE membrane (Jincheng Plastic, 1 μm thick, 19 × 8 cm) as the catalyst and a VUV lamp (185 nm, 14.9 cm × 1.5 cm) as the light source. Quartz was not used because it transmits VUV; a sealed configuration was adopted to minimize VUV leakage and ensure operational safety. Lamp gaps are sealed with AB-curing adhesive (3M DP110) to prevent moisture ingress; the UV lamp, posing radiation risks, is activated only after reaction initiation. The assembly is placed in an ultrasonic cleaner (Chunlin, 40 kHz, 120 W), with a peristaltic pump maintaining the water temperature at ~25 °C. For continuous operation, a 6-mm hole is drilled at the tube’s base, fitted with a 6-mm flexible gas tube secured by AB-curing adhesive. Two peristaltic pumps regulate liquid flow, with the output flow rate exceeding the input to ensure steady operation.

### Characterization

The UV–visible (UV–Vis) absorbance of the sample was measured using a PerkinElmer LAMBDA 1050+ UV–Vis spectrometer over a range of 250 to 600 nm. A 3-ml sample was placed in a quartz cuvette. SEM images and energy-dispersive x-ray analysis of samples were obtained using Zeiss GeminiSEM 500. Fourier transform infrared analysis was conducted using Bruker Vertex 70VISIBILITY over a range of 400 to 3,000 cm^−1^. The surface states were studied by x-ray photoelectron spectroscopy (Thermo Scientific ESCALAB Xi+, USA). EPR was recorded using Bruker EMX Plus-9.5/12/P/L. Measurements were conducted in the X-band (9.830243 GHz) with an amplitude modulation of 1 G, a microwave power of 2 mW, an amplitude modulation frequency of 100 kHz, and a conversion time of 60 ms.

### Indigo disulfonate method for the quantification of ozone

Indigo disulfonate was prepared as a 0.1 mM aqueous solution, and 10 ml of ozone water with previously quantified concentrations of 8.10, 4.05, 2.025, 1.0125, and 0 ppm was added. Then, 1 ml of 1 mM *N*,*N*-diethyl-*p*-phenylenediamine sulfate was added, and the solution was allowed to stabilize until the color was stable. The absorbance was measured at 512 nm using a UV–Vis spectrophotometer, and the ozone concentration in the water was determined based on the standard curve.

### Antibiotic degradation detection

The degradation products of SDZ, CIP, and TC were qualitatively analyzed using ultrahigh-performance liquid chromatography (UHPLC)–MS and UHPLC–tandem MS coupled with electrospray ionization. The analysis was performed on a Nexera UHPLC system (Shimadzu Corp., Kyoto, Japan) integrated with a Shimadzu 9300 quadrupole time-of-flight high-resolution mass spectrometer. Chromatographic separation was achieved using a Thermo Scientific Hypersil Gold column (1.9-μm particle size, 2.1 × 50 mm; Waltham, MA, USA) with water (mobile phase A) and acetonitrile (mobile phase B) in a volumetric ratio of 9:1. The flow rate was maintained at 0.3 ml/min under isocratic elution mode for 4 min. Mass spectrometric parameters included a time-of-flight mass scan range of *m*/*z* 10 to 500, an interface voltage of 4 kV, and a column oven temperature set to 35 °C.

The removal rate of organic pollutants (conversion efficiency) was calculated using the following equation:

$$C=\left(C_{o}-C_{t}\right)/C_{o}\times 100\%$$ (9)

where *C* (%) is the conversion rate, *C*_o_ is the concentration of the pollutants before treatment, and *C*_t_ represents the concentration of the pollutants after treatment. The degradation rate constant was evaluated by a pseudo-first-order kinetics model:

$$\ln\left(C_{o}/C_{t}\right)=k_{\mathrm{obs}}\times t$$ (10)

where *k*_obs_ is the degradation rate constant, *C*_o_ is the concentration of the pollutants before treatment, and *C*_t_ represents the concentration of the pollutants after treatment.

### Disinfection performance evaluation

ESKAPE pathogens ( *E. coli*, ATCC 25922; *S. aureus*, ATCC 29213; *A. baumannii*, ATCC 19606; *Enterococcus faecalis*, ATCC 29212; *K. pneumoniae*, ATCC 13883; and *P. aeruginosa*, ATCC 27853) were cultured in LB medium for 12 h at 37 °C to log phase and then washed with ultrapure water twice to remove the residues of the growth medium and resuspended to reach a final OD_600_ of 0.09 to 0.10 (10^8^ CFU/ml) for disinfection assay. Pathogen suspensions (untreated and treated with respective systems) were serially diluted (10^0^ to 10^−6^) in sterile water; 5 μl of each dilution was spotted onto LB agar plates, followed by incubation at 37 °C for 16 h. CFUs were quantified to assess bacterial viability.

### D_2_O-labeled single-cell Raman spectroscopy

Five hundred microliters of treated bacterial suspension with different conditions (CEC–VUV, VUV, and CEC) was mixed with 500 μl of 2× LB containing 50% heavy water (D_2_O) into sterilized tubes. The samples were incubated at 37 °C and 150 rpm for 12 h. All of the cells were harvested by centrifuging at 8,000 rpm for 3 min and then washed with sterile water twice to remove the culture medium. After washing, samples were spotted on an aluminum (Al) foil substrate and dried at room temperature prior to single-cell Raman spectral acquisition. Single-cell Raman spectroscopy was performed using a LabRAM Aramis (HORIBA LabRAM Odyssey, Japan) confocal micro-Raman system equipped with a 532-nm Nd:YAG excitation laser and a 300 grooves/mm diffraction grating. A 100× dry objective (numerical aperture = 0.9, Olympus, Japan) was employed for bacterial observation and spectral acquisition. The spectra were processed by baseline correction and normalization in the LabSpec 5 software (HORIBA Jobin-Yvon, Japan). The bands assigned to CD (2,040 to 2,300 cm^−1^) and CH (2,800 to 3,100 cm^−1^) were integrated to calculate the ratio of C_D_/(C_D_ + C_H_) to indicate the deuterium incorporation extent.

## Data Availability

The data supporting this study’s findings are available within the article and its supplementary materials.
