# Supplementary material for: Additive-Free Contact-Electro-Catalysis/Vacuum Ultraviolet System for Rapid Mitigation of Antimicrobial-Resistance-Associated Contaminants in Water
Source: Research (Wash D C). 2026 Jul 1;9:1348. doi: 10.34133/research.1348 (PMC13319629; doi:10.34133/research.1348)
Supplement: Supplementary 1 — Supplementary Methods Figs. S1 to S29 Tables S1 to S4 [file research.1348.f1.docx]

**Additive-Free Contact-Electro-Catalysis/Vacuum Ultraviolet System for Rapid Mitigation of Antimicrobial Resistance-Associated Contaminants in Water**

Weixin Li^1^†, Xinyu Xing^2,4^†, Kai Yang^2^†, Jikai Sun^3^, Jialuo Tu^1^, Zihan Liang^1^, Fang Cao^1^, Shanshan Wang^1^, Jianing Dong^1^, Li Cui^2^*, Yong-Guan Zhu^2,4^, Zhong-Qun Tian^1^, Feng Ru Fan^1^*

^1^State Key Laboratory of Physical Chemistry of Solid Surfaces, iChEM, College of Chemistry and Chemical Engineering, Innovation Laboratory for Sciences and Technologies of Energy Materials of Fujian Province (IKKEM), Xiamen University, Xiamen 361005, China.

^2^State Key Laboratory for Ecological Security of Regions and Cities, Institute of Urban Environment, Chinese Academy of Sciences, Xiamen, 361021, China.

^3^Department of Chemical and Environmental Engineering, University of California, Riverside, California 92521, United States.

^4^State Key Lab of Urban and Regional Ecology, Research Center for Eco-Environmental Sciences, Chinese Academy of Sciences, Beijing 100085, China.

†These authors contributed equally to this work

*Corresponding author. E-mail: lcui@iue.ac.cn, frfan@xmu.edu.cn

**The PDF file includes:**

Methods

Figs. S1 to S23

Tabs. S1 to S3

**Methods**

**Chemicals and materials**

Sodium chloride (NaCl, ACS, ≥99%) from Sinopharm, sodium sulfate (Na_2_SO_4,_ ACS, ≥99%) from Sinopharm, sodium nitrate (NaNO_3_, ACS, ≥99%) from Sinopharm, sodium dihydrogen phosphate (NaH_2_PO_4_, AR, 99%) from Sinopharm, tertiary butanol (TBA, AR, ≥99.5%) from Sinopharm, furfuryl alcohol from Aladdin (AR, ≥99%), methanol (AR, ≥99%) from Sinopharm, DMPO (5,5-dimethyl-1-pyrroline N-oxide, 97%) from Energy Chemical, and TEMP (2,2,6,6-Tetramethyl-4-piperidone hydrochloride) from Aladdin. Benzoic acid from Aladdin (BA, ACS, 99.5%), titanium potassium oxalate (ACS, 98%) from Aladdin, sulfadiazine (SDZ) from Macklin, ciprofloxacin (CIP) from Macklin, tetracycline hydrochloride (TC) from Aladdin, lysogeny broth from HOPEBIO, propidium Iodide from InvivoChem, 2xTaq Master Mix (Dye Plus) from Vazyme；DL2000 PIus DNA Marker from Vazyme, and D_2_O (99.0%) from Sigma.

**Catalytic reactions**

The reaction setup comprises a glass tube (20 cm long, 3 cm diameter) housing a commercial PTFE membrane (Jincheng Plastic, 1 μm thick, 19 × 8 cm) as the catalyst and a vacuum UV lamp (185 nm, 14.9 cm × 1.5 cm) as the light source. Quartz glass is avoided due to UV penetration hazards. Lamp gaps are sealed with AB-curing adhesive (3M DP110) to prevent moisture ingress; the UV lamp, posing radiation risks, is activated only after reaction initiation. The assembly is placed in an ultrasonic cleaner (Chunlin, 40 kHz, 120 W), with a peristaltic pump maintaining the water temperature at ~25°C. For continuous operation, a 6-mm hole is drilled at the tube’s base, fitted with a 6-mm flexible gas tube secured by AB-curing adhesive. Two peristaltic pumps regulate liquid flow, with the output flow rate exceeding the input to ensure steady operation.

**Characterization**

The UV-Vis absorbance of the sample was measured using a PerkinElmer LAMBDA 1050+ UV-Visible spectrometer over a range of 250–600 nm. A 3 mL sample was placed in a quartz cuvette. Scanning electron microscopy (SEM) images and Energy Dispersive X-Ray analysis (EDX) of samples were obtained using a Zeiss Gemini-SEM 500. FTIR analysis was conducted using a Bruker Vertex 70VISIBILITY over a range of 400–3000 cm^-1^. The surface states were studied by X-ray photoelectron spectroscopy (XPS, Thermo Scientific ESCALAB Xi+, USA). Electron paramagnetic resonance (EPR) was recorded using a Bruker EMX Plus-9.5/12/P/L. Measurements were conducted in X-band (9.830243 GHz) with an amplitude modulation of 1 G, microwave power of 2 mW, amplitude modulation frequency of 100 kHz, and conversion time of 60 ms.

**IDS method for quantification of ozone**

IDS was prepared as a 0.1 mM aqueous solution, and 10 mL of ozone water with previously quantified concentrations of 8.10, 4.05, 2.025, 1.0125, and 0 ppm was added. Then, 1 mL of 1 mM DPD was added, and the solution was allowed to stabilize until the color was stable. The absorbance was measured at 512 nm using a UV-visible spectrophotometer, and the ozone concentration in the water was determined based on the standard curve.

**Antibiotic degradation detection**

The degradation products of sulfadiazine (SDZ), ciprofloxacin (CIP), and tetracycline (TC) were qualitatively analyzed using ultrahigh-performance liquid chromatography-mass spectrometry (UHPLC-MS) and ultrahigh-performance liquid chromatography-tandem mass spectrometry (UHPLC-MS/MS) coupled with electrospray ionization (ESI). The analysis was performed on a Nexera UHPLC system (Shimadzu Corp., Kyoto, Japan) integrated with a Shimadzu 9300 Q-ToF high-resolution mass spectrometer. Chromatographic separation was achieved using a Thermo Scientific Hypersil Gold column (1.9 μm particle size, 2.1 × 50 mm; Waltham, MA, USA) with water (mobile phase A) and acetonitrile (mobile phase B) in a volumetric ratio of 9:1. The flow rate was maintained at 0.3 mL/min under isocratic elution mode for 4 min. Mass spectrometric parameters included a TOF mass scan range of m/z 10–500, an interface voltage of 4 kV, and a column oven temperature set to 35 °C.

The removal rate of organic pollutants (conversion efficiency) was calculated using the following equation:

C = (C_o_ – C_t_)/C_o_ × 100% (3)

where C (%) is the conversion rate, C_o_ is the concentration of the pollutants before treatment, Ct represents the concentration of the pollutants after treatment. The degradation rate constant was evaluated by a pseudo first-order kinetics model:

ln(C_o_/C_t_) = k_obs_ × t (4)

where k_obs_ is the degradation rate constant, C_o_ is the concentration of the pollutants before treatment, C_t_ represents the concentration of the pollutants after treatment.

**Disinfection Performance Evaluation**

ESKAPE Pathogens (Escherichia coli, E.coli, ATCC25922; Staphylococcus aureus, S.aureus, ATCC29213; Acinetobacter baumannii, A.baumannii, ATCC19606; Enterococcus faecalis, E.faecalis, ATCC29212; Klebsiella pneumoniae, K.pneumoniae, ATCC13883 Pseudomonas aeruginosa, P. aeruginosa ATCC27853) were cultured in Luria-Bertani (LB) for 12 h at 37 °C to log phase and then washed with UP water twice to remove the residues of growth medium and re-suspended to reach the final OD600 of 0.09–0.10 (108 colony-forming unit/mL, CFU/mL) for disinfection assay. Pathogen suspensions (untreated and treated with respective systems) were serially diluted (10⁰ to 10⁻6) in sterile water. 5 μL of each dilution were spotted onto LB agar plates, followed by incubation at 37°C for 16 h. Colony-forming units (CFUs) were quantified to assess bacterial viability.

**D_2_O-Labeled Single-Cell Raman Spectroscopy**

500 μL of treated bacterial suspension with different conditions (CEC-VUV, VUV, CEC) was mixed with 500 μL of 2×LB containing 50% heavy water (D_2_O) into sterilized tubes. The samples were incubated at 37 ^o^C, 150 rpm for 12 h. All of the cells were harvested by centrifuging at 8000 rpm for 3 min and then washed with sterile water twice to remove the culture medium. After washing, samples were spotted on aluminum (Al) foil substrate and dried at room temperature prior to single-cell Raman spectral acquisition. Single-cell Raman spectroscopy was performed using a LabRAM Aramis (HORIBA LabRAM Odyssey, Japan) confocal micro-Raman system equipped with a 532 nm Nd:YAG excitation laser and a 300 grooves/mm diffraction grating. A 100× dry objective (N.A. = 0.9, Olympus, Japan) was employed for bacterial observation and spectral acquisition. The spectra were processed by baseline correction and normalization in LabSpec 5 (HORIBA Jobin-Yvon, Japan) software. The bands assigned to CD (2040−2300 cm^−1^) and CH (2800−3100 cm^−1^) were integrated to calculate the ratio of CD/(CD + CH) to indicate the deuterium incorporation extent.

**Reactive oxygen species (ROS) analysis**

Electron paramagnetic resonance (EPR) testing was performed at room temperature using a sample containing 50 mL deionized (DI) water, a PTFE film, and 100 µL DMPO. Hydroxyl radicals (^•^OH) was trapped by DMPO, and singlet oxygen (^1^O_2_) were trapped by TEMP. Changes in free radical production after 10 minutes of ultrasonication were compared.

**Quantitative Characterization of Hydrogen Peroxide (H_2_O_2_)**

First, a 1000 ppm hydrogen peroxide standard solution was diluted to various concentrations in 2 mL aliquots, and 2 mL of 0.1 M titanium potassium oxalate (C_4_H_2_K_2_O_10_Ti) solution was added. A 4 mL sample of the mixed solution was analyzed for UV-Vis absorbance at 385 nm, and a standard curve was generated based on the corresponding hydrogen peroxide concentrations.

^•^**OH Detection Using Coumarin as Probe**

Coumarin was employed as a fluorescent probe to monitor ^•^OH generation. When coumarin reacts with ^•^OH, it forms 7-hydroxycoumarin (7-HC). The fluorescence of 7-HC was measured using a fluorescence spectrometer (Edinburgh FLS1000) with an excitation wavelength of 350 nm and an emission wavelength of 450 nm.

**Quantitative Analysis of Cumulative ^•^OH Concentration**

The cumulative ^•^OH concentration was determined via a fluorescence-based method using benzoic acid as a chemiluminescent probe. The resulting p-hydroxybenzoic acid (p-HBA) was quantified using a High-Performance Liquid Chromatography (HPLC) system equipped with SPD15C UV-Vis detector (detection wavelength: 225 nm), a WondaCract ODS-2 column (250 mm × 4.6 mm × 5 μm), and a mobile phase of acetonitrile/0.1% (v/v) H_3_PO_4_ (4:6 ratio, v/v) at a flow rate of 1 mL/min. The retention time of p-HBA under these conditions was approximately 0.53 minutes.

**Environmental water sampling**

Seawater was sourced from Baicheng Beach, Xiamen, China (24°43’ N, 118°10’ E), and bay water from Xinglin Bay, Xiamen, China (24°36’ N, 118°03’ E).

**Fluorescence microscopy and Flow Cytometry Fluorescence Detection**

1 mL ESKAPE pathogen suspensions before/after treatment was added 1 μL of 5 mM propidium iodide DMSO solution (PI; InvivoChem ) and1 μL of 5 mM SYTO9 to achieve a final dye concentration of 5 μM. The samples were incubated for 10 min at room temperature in the dark and then washed three times with 1× PBS. then applied to a microscope slide for analysis. The stained bacterial samples were examined using a laser scanning confocal microscope (LSCM; LSM900, Olympus). Stained cells were analyzed using a flow cytometer (guava easycyte 5 HLP) equipped with a 488-nm laser. Fluorescence emission was collected through a 585/50 nm bandpass filter (FL3 channel for PI). Data from 5,000 events per sample were acquired. Data were processed with FlowJo v10.8 software.

**Scanning Electron Microscopy (SEM).**

The ESKAPE Pathogens before (intact bacteria) and after (damaged bacteria) treatment were characterized by SEM (Hitachi S4800, Japan). The samples were fixed with 2.5% glutaraldehyde for 12 hours at 4 °C. The fixed samples were then dehydrated using solutions with increasing concentrations of ethanol (30%, 50%, 70%, 90% and 100%; v/v) for 10 min successively, followed by a carbon dioxide critical point drying process (Autosamdri 815 automatic critical point dryer apparatus; Tousimis, USA) overnight for SEM characterization. The samples were adhered to the conductive tapes (SPI, USA) mounted on a copper stub and sputter coated with gold for SEM analysis.

**DNA Extraction and Gel electrophoresis analysis**

DNA was extracted from water samples with different reaction conditions using the FastDNA Spin Kit for Soil (MP Biomedicals, Santa Ana, USA) according to the manufacturer’s guidelines. In total, 600 mL water samples were used for DNA extraction in triplicate. Water samples were filtered through sterile 0.22 μm polycarbonate filters (Merck Millipore Ltd., Ireland), which were then cut into pieces using sterile scissors and subjected to DNA extraction. The concentration of DNA was quantified using a QuantiFluor dsDNA assay kit (Promega, WI, USA) with a Qubit 3.0 Fluorometer (Thermo Fisher Scientific Inc., Waltham, USA). Finally, the DNA extracts of all samples were stored at −20 °C until further analysis.

**Determination of 16S rRNA fragments of different lengths, ARGs and integron gene**

To quantify the relative abundances of 1400-bp and 40-bp 16S rRNA fragments, antibiotic resistance genes (sul1, tetA), and the integron gene (intl1) in environmental DNA (eDNA) before and after treatment in Xinglin Bay, The qPCR assay was conducted using SYBR Green SuperReal PreMix Plus (TIANGEN Biotech Corp., China) in a Real Time PCR System (Roche LightCycler480II Germany). The sequences of primers are summarized in Table S3.

**Cost calculation**

The PTFE membrane, measuring 0.1 cm in thickness, 200 cm in width, and 1000 cm in length, was commercially procured from Taobao.com at a price of USD 13.77. For a single operational unit (0.1 cm × 200 cm × 5 cm), the material cost was calculated as USD 0.069. The CEC-VUV system exhibited exceptional sterilization performance, maintaining >99.999% efficiency at a flow rate of 1150 mL/min, with energy consumption parameters based on the U.S. average industrial electricity rate of USD 0.08/kWh. The system’s power usage of 0.11 kWh per cycle resulted in an electricity cost of USD 1.594 per kWh·m^-3^, resulting in a total operational cost of USD 0.197 per cubic meter, including material costs.

**Theoretical calculation simulation**

In this study, density functional theory (DFT) calculations were performed using the Dmol3 code.^1^ The exchange-correlation interactions were described using the generalized gradient approximation (GGA) with the PBE functional.^2^ A double numerical quality basis set with d-type polarization functions (DNP)^3^ was employed for all geometric optimizations and total energy calculations. The core electrons were modeled using effective core pseudopotentials (ECP) developed by Dolg^4^ and Bergner^5^. All calculations were carried out using a spin-unrestricted framework. To simulate the solvent environment, the conductor-like screening model (COSMO)^6^ with a dielectric constant of 78.54 was applied. The positions of all atoms were fully relaxed until the following convergence criteria were satisfied: a force threshold of 0.002 Ha/Å, a total energy convergence of 10^-5^ Ha, and a displacement limit of 0.005 Å. A real-space cutoff radius of 4.4 Å was used, and the self-consistent field (SCF) calculations were converged to a threshold of 10^-6^ Ha.

**
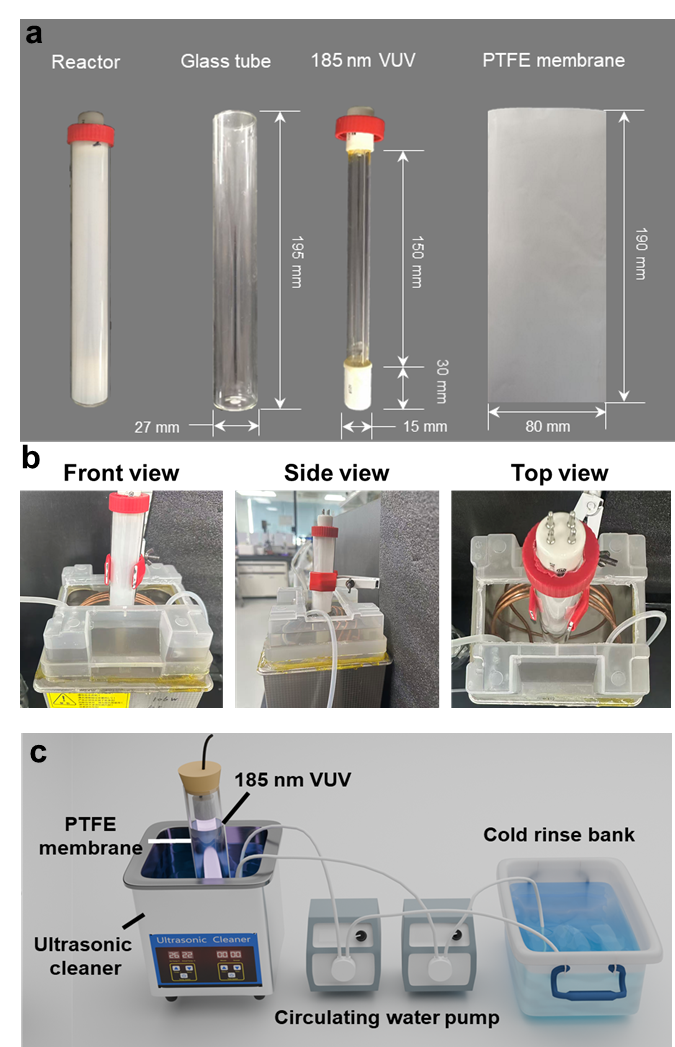
**

**Fig. S1. Schematic diagram of the reaction apparatus.** (a) Reactor assembly. Includes glass tubing, 185 nm VUV, and PTFE membrane (b) Three-view diagram of the reactor. (c) Overall reaction diagram. Including ultrasonic reactor, light source, circulating water pump and cold-water tank.

**
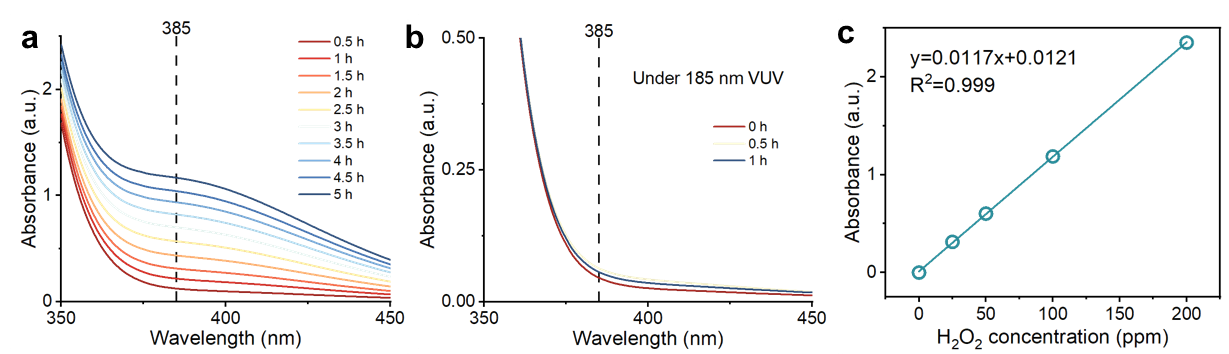
 Fig. S2. Quantification of H_2_O_2_ concentration using potassium titanium oxalate (PTO) method.** (a) Every 30 minutes over 5 hours, 2 mL of solution was sampled, mixed with 2 mL of 0.1 M titanium potassium oxalate, and analyzed for absorbance at 385 nm. (b) Absorbance at 385 nm VUV irradiation in the CEC system. (c) Calibration curve of absorbance at 385 nm versus known H_2_O_2_ concentrations.


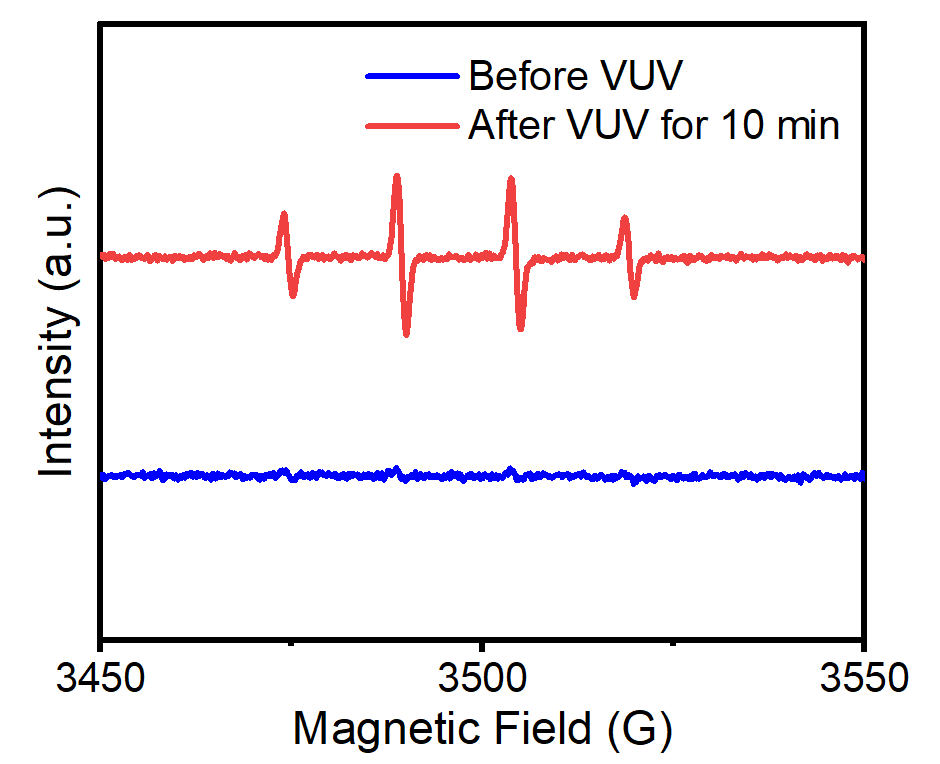


**Fig. S3.** **EPR detection of ^•^OH signals before and after VUV exposure in the presence of 20 ppm** **H_2_O_2_.** 20 ppm H_2_O_2_ and 100 μL of DMPO (as a ^•^OH trapping agent) were added to 50 mL of water. Before VUV irradiation, 1 mL of solution was sampled; after 10 minutes of VUV exposure, another 1 mL was sampled for EPR analysis. VUV irradiation decomposed H_2_O_2,_ forming ^•^OH radicals, resulting in a DMPO-^•^OH signal.


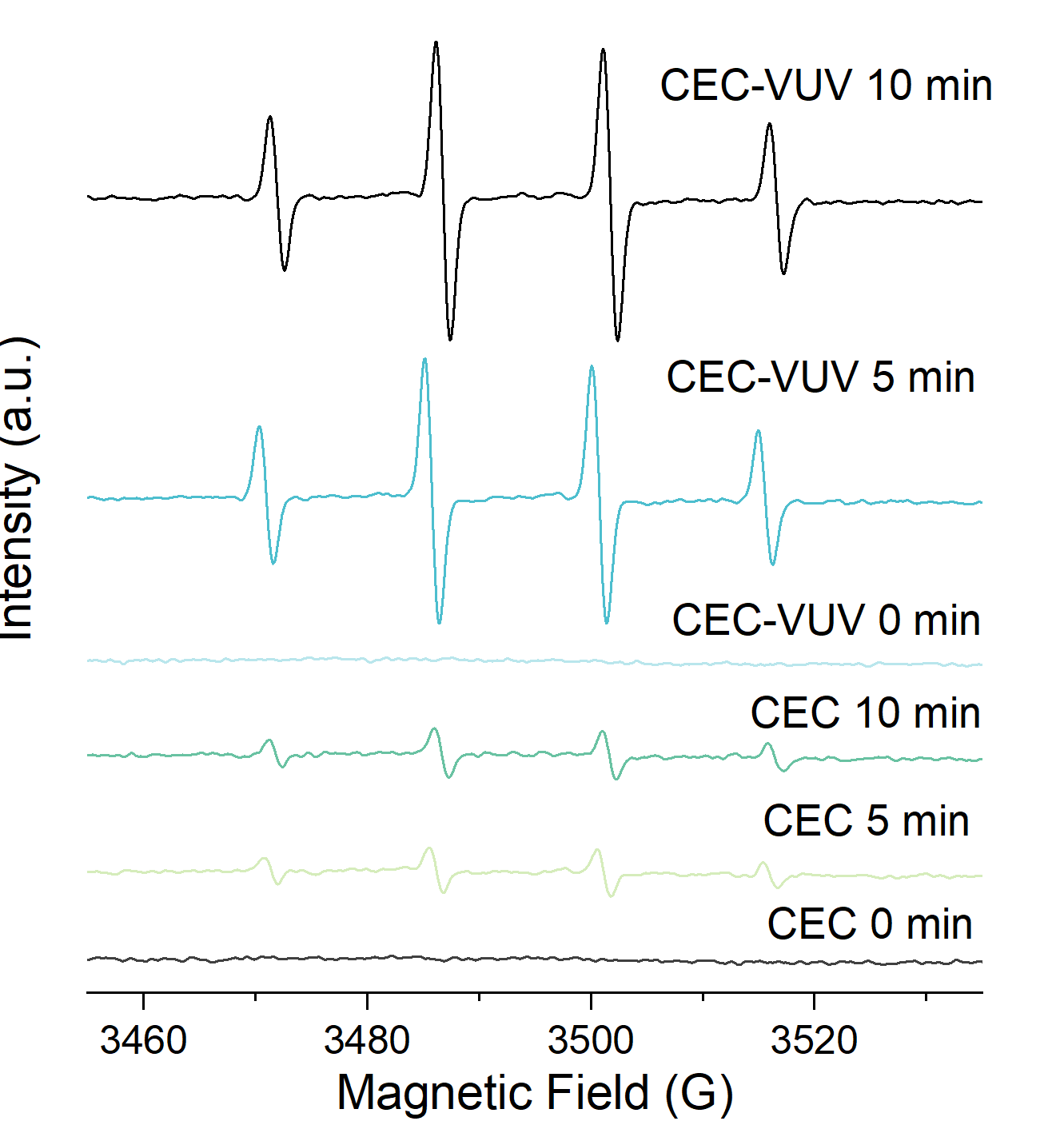


**Fig. S4. EPR data of the CEC and CEC-VUV systems at different reaction time.** The results show that the characteristic signal of ^•^OH continuously increased with reaction time, indicating that ^•^OH generation in this system is sustained and accumulative rather than occurring as a transient pulse.


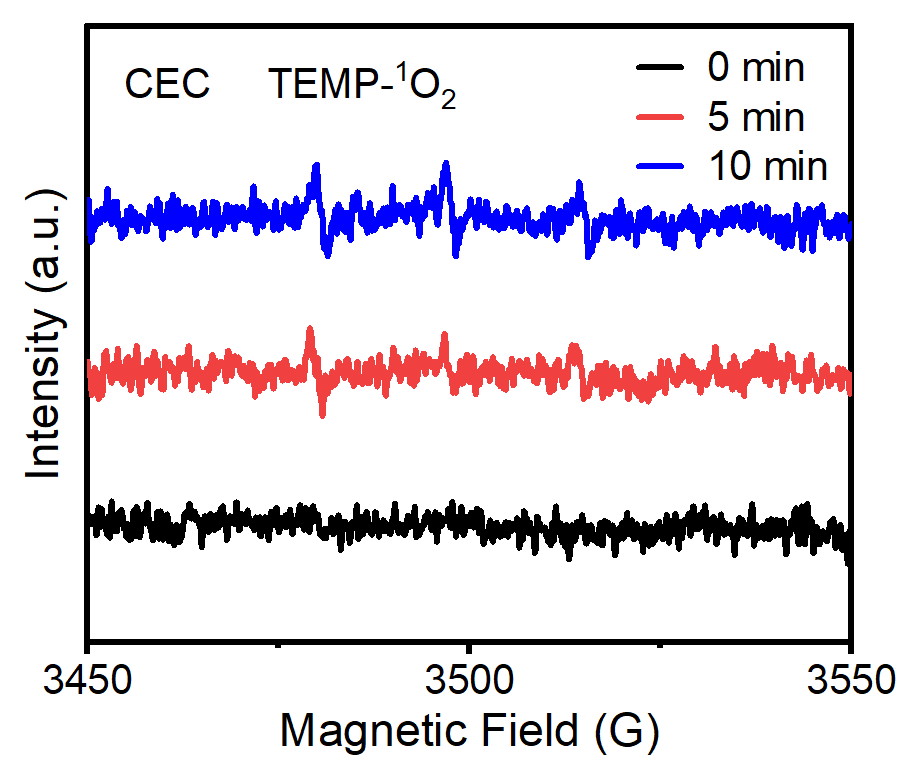


**Fig. S5. EPR detection captures singlet oxygen generated by CEC through TEMP.** The signal intensity was relatively weak which is not primarily due to the use of PTFE membranes instead of PTFE particles, but rather to reactor configuration and oxygen availability.


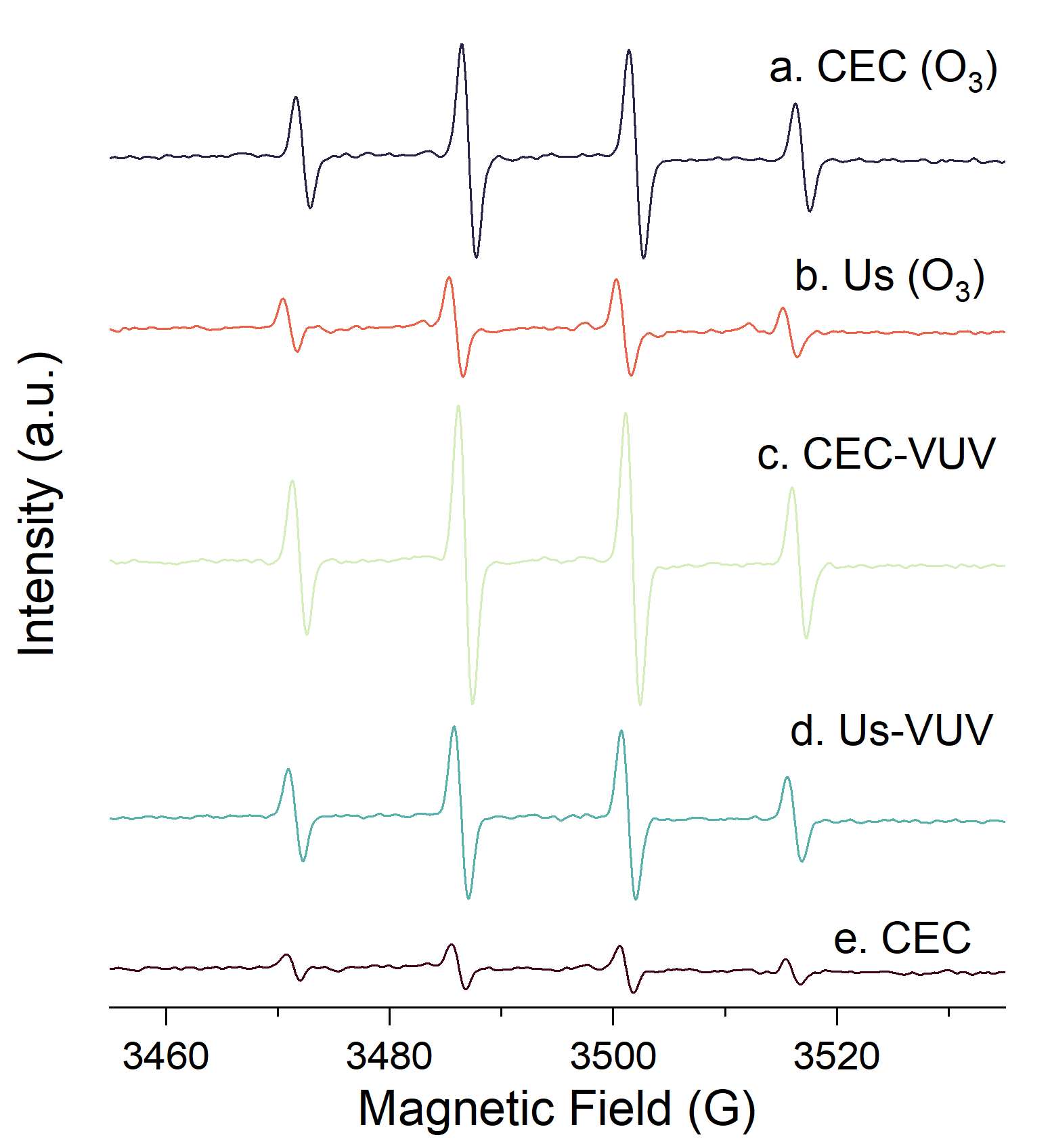


**Fig S6. Comparative EPR experiments were performed under different reaction conditions.** The experimental setup consisted of 5 mL of 1 mM DMPO solution as the spin-trapping system, with 5 mg of PTFE powder added when required. The specific conditions were as follows: Control group a: 5 mL of 1 mM DMPO solution + 5 mg PTFE, pre-irradiated with VUV for 10 min, followed by ultrasound for 10 min. Control group b: 5 mL of 1 mM DMPO solution, pre-irradiated with VUV for 10 min, followed by ultrasound for 10 min. Control group c: 5 mL of 1 mM DMPO solution + 5 mg PTFE, with VUV and ultrasound applied simultaneously for 10 min. Control group d: 5 mL of 1 mM DMPO solution, with VUV and ultrasound applied simultaneously for 10 min. Control group e: 5 mL of 1 mM DMPO solution + 5 mg PTFE, treated with ultrasound alone for 10 min.

The EPR results showed that a distinct ^•^OH signal could already be detected in control group e, indicating that the system itself is capable of generating ^•^OH under CEC conditions. Comparison between control groups c and d further showed that ^•^OH could still be produced under the combined action of VUV and ultrasound even in the absence of PTFE, whereas the ^•^OH signal was significantly enhanced after the addition of PTFE, demonstrating that CEC can markedly promote ^•^OH generation. Further comparison among control groups a, b, and e revealed that, after VUV pre-irradiation, the O_3_ generated in the system could further participate in ^•^OH production under subsequent ultrasonic conditions, and this signal became stronger in the presence of PTFE, indicating that CEC facilitates O_3_ activation and its conversion into ^•^OH. Combined with the quantitative results of H_2_O_2_ and O_3_ presented above, these supplementary experiments collectively support the following conclusion: VUV promotes the conversion of H_2_O_2_ into ^•^OH, while CEC facilitates O_3_ activation and its further transformation into ^•^OH.


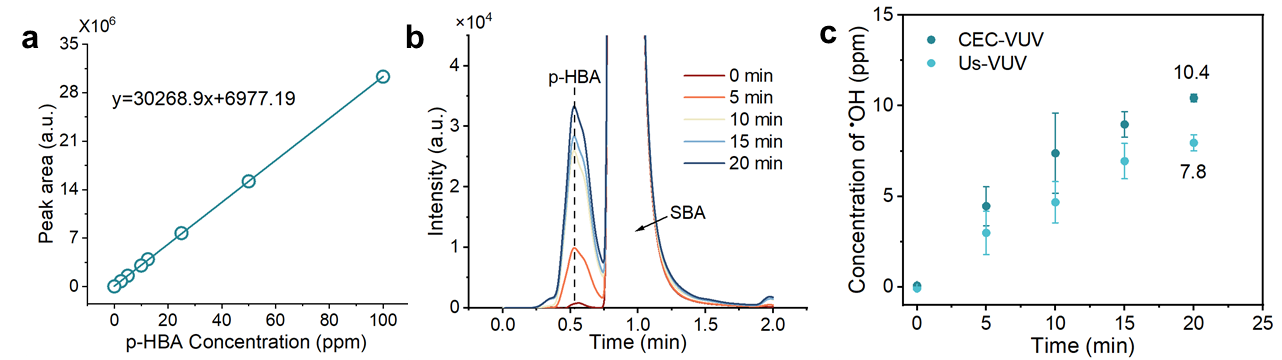


**Fig. S7. Quantification of ^•^OH generation rate using sodium benzoate (SBA) as a probe.**

To quantify the generation rate of ^•^OH, SBA was employed as a probe. (a) Quantitative calibration curve of p-HBA concentration versus integrated peak area. (b) HPLC signals: SBA at different reaction times and the product p-HBA. (c) Using p-HBA as a ^•^OH yield probe, comparison of the ^•^OH concentrations generated by contact-electrification–photo coupling and ultrasound–photo coupling. SBA reacts with OH through a hydroxylation reaction to produce a fluorescent product, p-hydroxybenzoic acid (p-HBA), which serves as an indicator for the accumulated ^•^OH concentration. An excess amount of the ^•^OH probe (10 mM, 0.721 g in 500 mL) was used to ensure complete capture of all photogenerated ·OH radicals. It is assumed that photolysis or oxidative degradation of p-HBA during the reaction is negligible. Therefore, all generated ^•^OH is considered to be quantitatively captured by SBA.


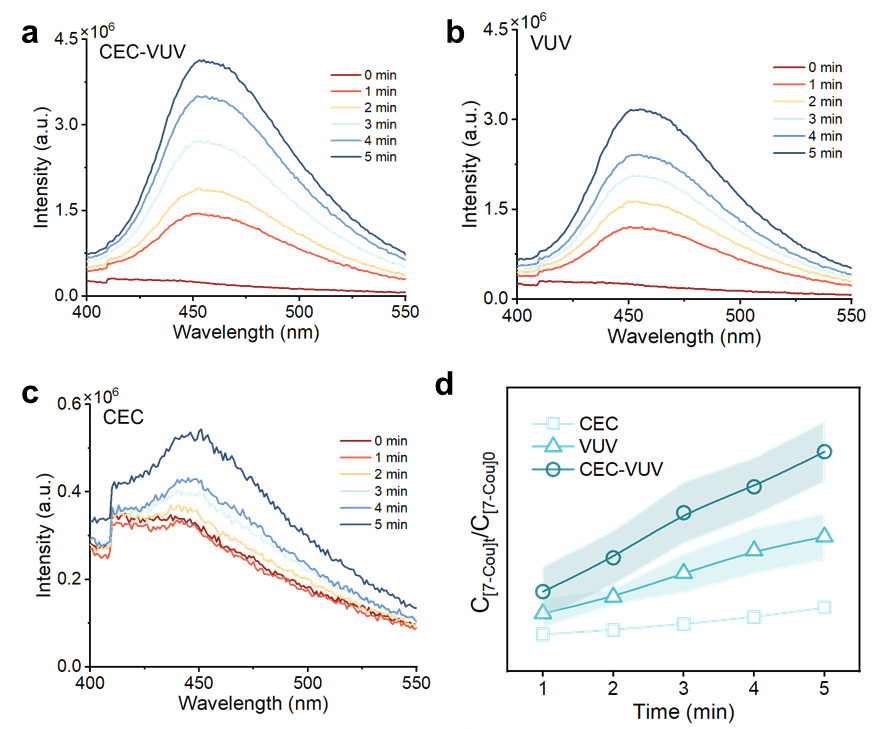


**Fig. S8. Detection of 7-HC formation via fluorescence spectroscopy under different reaction systems.** (a) CEC-VUV. (b) VUV (c) CEC. (d) Linear relationship of 7-HC fluorescence intensity over time in different systems.

Fluorescence emission at 450 nm was monitored to detect the formation of 7-Cou, the reaction product of coumarin and ^•^OH, under various reaction conditions. The coumarin concentration in the solution was 1000 ppm. A 3 mL sample was taken every 1 minute for a total duration of 5 minutes. Since coumarin itself may be partially affected by VUV irradiation, precise quantification was not performed. Instead, this method was used as a supplementary approach to support the quantitative results of ^•^OH concentrations obtained using the sodium benzoate method.


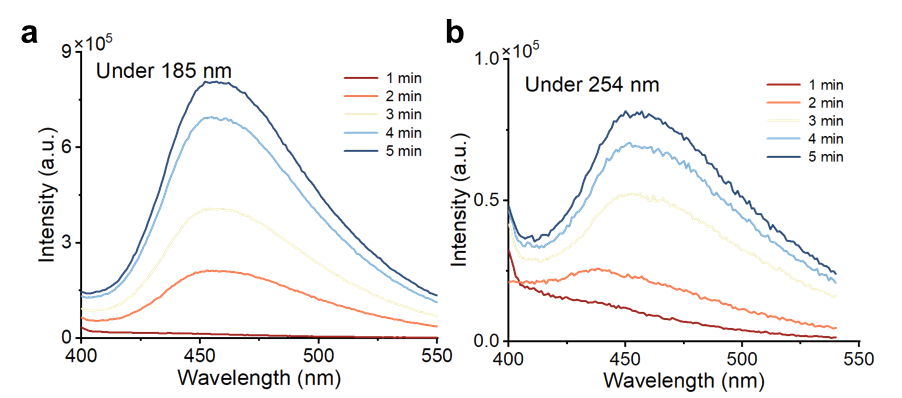


**Fig. S9. Fluorescence spectroscopy was used to detect the emission peak of 7-HC at 450 nm.** Forming from the reaction between coumarin and ^•^OH in the CEC system under different irradiation wavelengths of (a) 185 nm and (b) 254 nm.


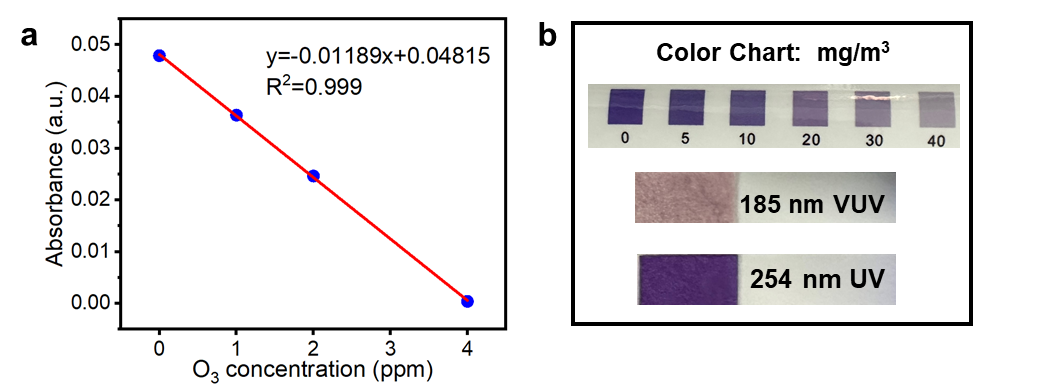


**Fig. S10.** **Standard curve of dissolved ozone concentration.** (a) Standard curve of O_3_ concentration in water. (b) Estimation of O_3_ concentration generated in the gas phase by 185 nm and 254 nm UV irradiation using standard detection strips.

Take 40 mL of deionized water, aerate it with O_3_ gas (ozone generator, pure oxygen as the inlet) for about 30 minutes, and add 0.6 mL of sulfuric acid (H_2_SO_4_) with a concentration of 0.0857 mol/L (1+6 mL DI) to adjust its pH <3. Next, measure the concentration of the solution using the following steps: add 20 mL of a solution containing 1 g of sodium iodide (NaI), titrate with 0.05 mol/L of sodium thiosulfate (Na_2_S_2_O_3_), add an appropriate amount of starch indicator to turn the solution blue until the color changes to light yellow, and then continue to titrate with 0.05 mol/L of Na_2_S_2_O_3_ until the solution fades, and record the volume of 0.05 mol/L Na_2_S_2_O_3_ used. The final volume of sodium thiosulfate titrated is 270 μL, and the concentration of dissolved ozone in water is measured to be 8.10 ppm.

2 C(O_3_)×V(O_3_) = C(Na_2_S_2_O_3_)×V(Na_2_S_2_O_3_) (1)

C(O_3_)=270×10^-6^×0.05/40×10^-3^ = 3.375×10^-4^ mol/L = 48 g/mol×0.3375 = 8.1 mg/L (2)

**Table S1.** Summary of control experiments and their mechanistic purposes.

| **Control system** | **Components** | **Purpose** | **Mechanistic implication** |
| --- | --- | --- | --- |
| CEC | PTFE + US | Evaluate PTFE-mediated contact electrification | Baseline ROS generation from CEC |
| VUV | VUV only | Evaluate VUV-induced ROS/O_3_ generation | Contribution of VUV without CEC |
| CEC–VUV | PTFE + US + VUV | Evaluate synergy | Coupled charge–photon ROS amplification |
| CEC–UV | PTFE + US + 254 nm UV | Exclude 185 nm-induced O_3_ pathway | Importance of VUV/O_3_ route |
| US–VUV | US + VUV without PTFE | Exclude PTFE-mediated CEC | Importance of PTFE/contact electrification |
| VUV/H_2_O_2_ | VUV + external H_2_O_2_ | Compare external and in situ H_2_O_2_ | Role of interfacial H_2_O_2_ generation |


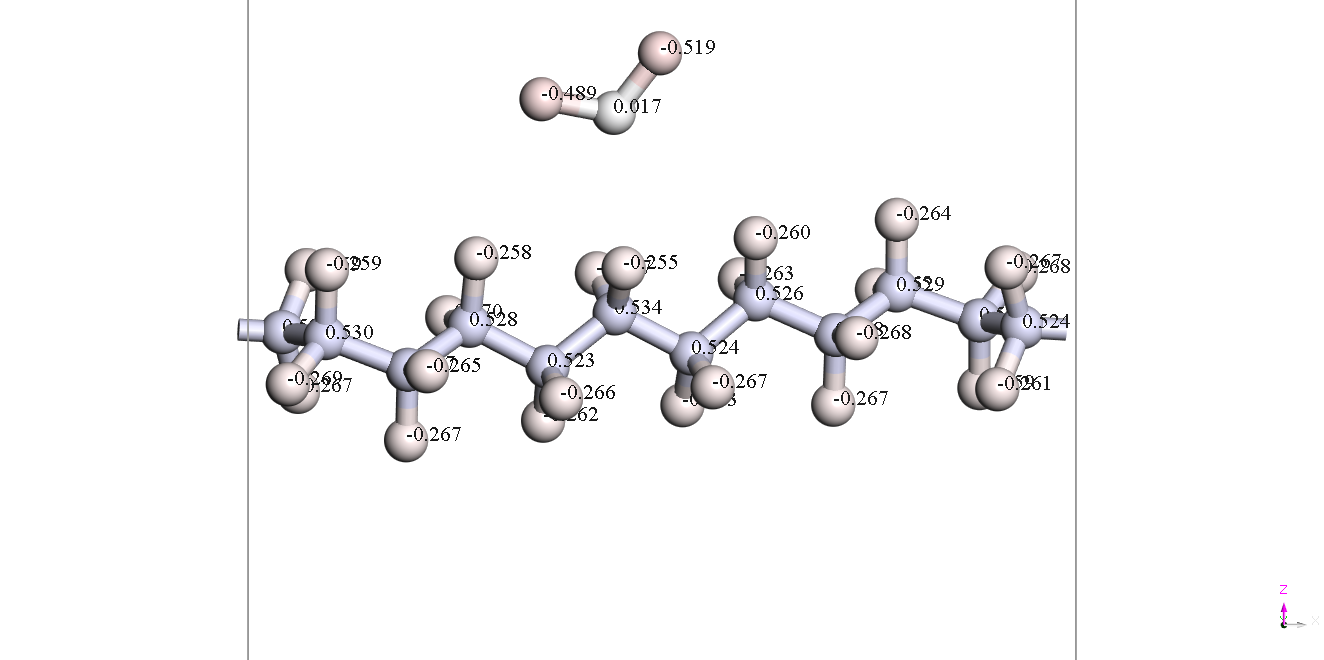


**Fig. S11. The Mulliken charge distribution after electron transfer.**

When PTFE^-^ comes into contact with O_3_, spontaneous electron transfer occurs. O_3_ carries a total charge of -0.991 e (out of -1 e in total), indicating that PTFE carries only -0.009 e, which suggests that nearly all the transferred electron density is localized on O_3_.


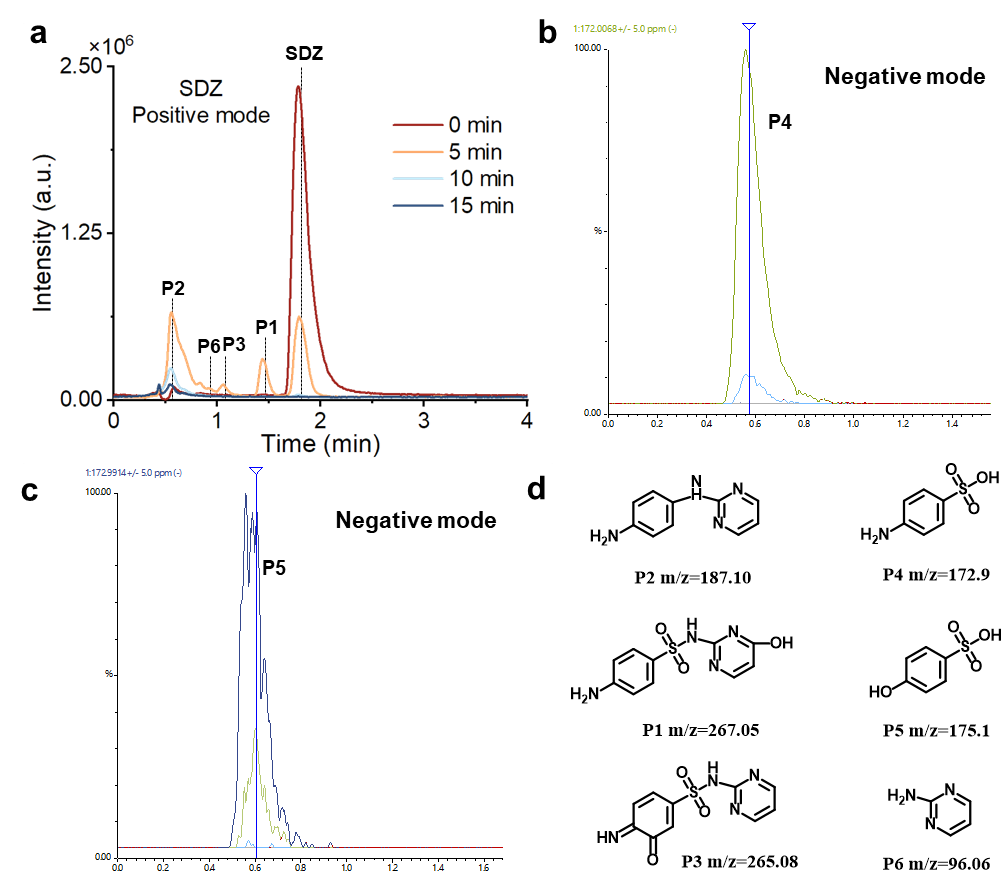


**Fig. S12. Analysis of SDZ degradation products by HPLC-MS.** (a) Signal peaks of SDZ and its degradation products in positive mode. (b) and (c) Signal peaks of SDZ degradation products in negative mode. (d) Summary of signal peaks and molecular structures of degradation products in both positive and negative modes.

In the positive mode, the signal peak at 1.8 min belonging to SDZ disappeared at 10 min, indicating that the degradation was complete, and the degradation intermediate signals P1, P2, P3 and P6 were detected at 5 min. In the negative mode, the intermediate signal peaks of P4 and P5 were detected.


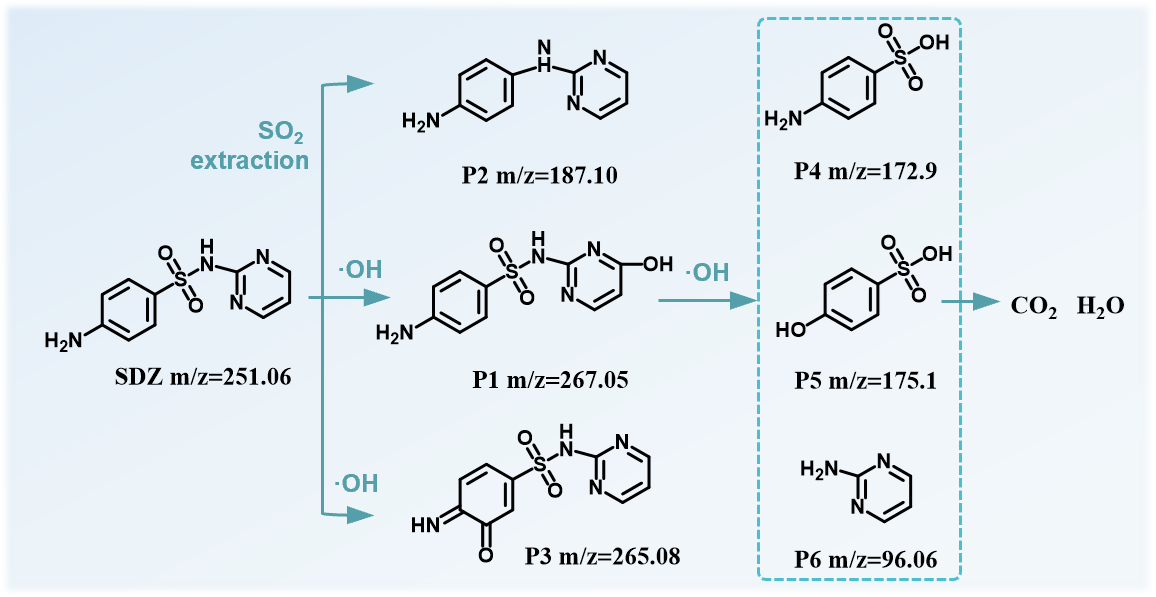


**Fig. S13. SDZ degradation pathway inferred by HPLC-MS.**


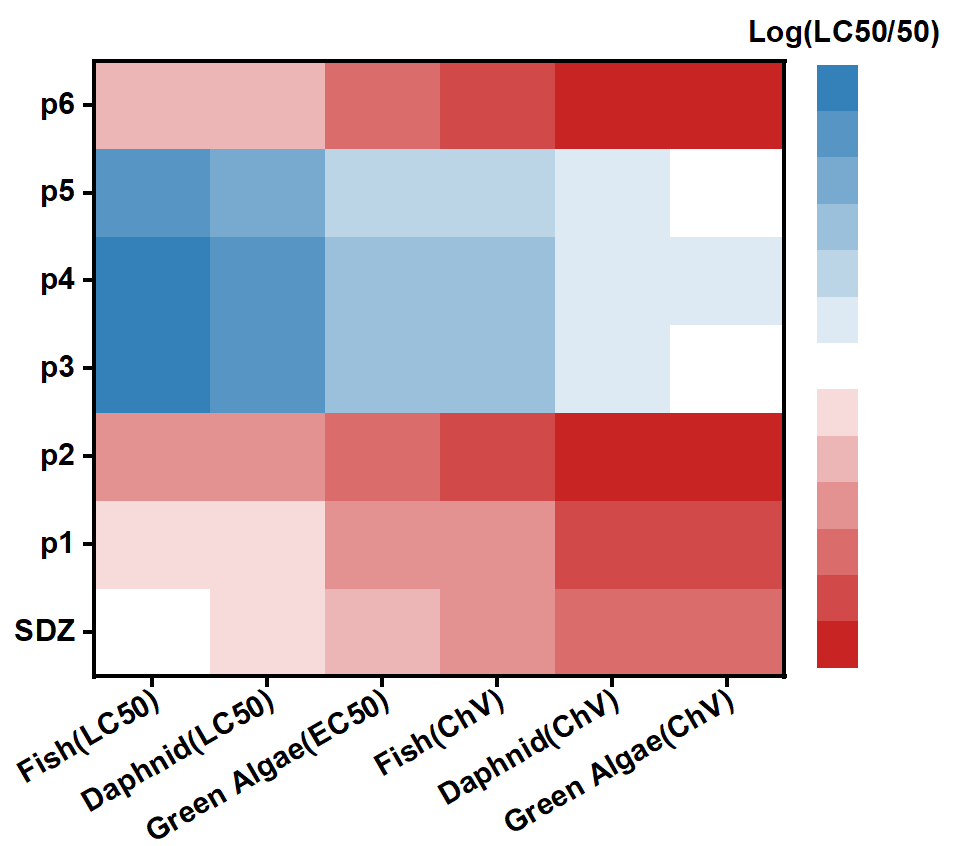


**Fig. S14. Toxicity variation of SDZ and its degradation intermediates.**

The toxicity variation of SDZ and its degradation intermediates was analyzed using the Ecosar software. Six indicators were selected, including the concentration that causes 50% mortality in fish within 96 hours (Fish (LC50)), the concentration that causes 50% mortality in daphnids within 48 hours (Daphnid (LC50)), the concentration that inhibits 50% algae growth rate within 96 hours (Green Algae (EC50)), the concentration that shows no visible harmful effects on fish under long-term exposure (Fish (ChV)), the concentration with no effect on daphnid reproduction under long-term exposure (Daphnid (ChV)), and the concentration with no inhibition on algae growth under long-term exposure (Green Algae (ChV)). The relevant results are shown in the Figure S9-10. In the early stages of degradation, the main products P1 and P2 exhibited significantly higher toxicity to fish, daphnids, and green algae compared to SDZ, whereas P3 had lower toxicity than SDZ. As the reaction proceeded, although P6 still showed some toxicity, the toxicity of P4 and P5 was significantly lower than that of P1, P2, and the original SDZ, indicating that with the progress of the reaction, SDZ was gradually degraded into low-toxicity small molecules and eventually mineralized completely. Furthermore, no corresponding intermediates were detected in the final reaction products or during the continuous reaction process by HPLC-MS.


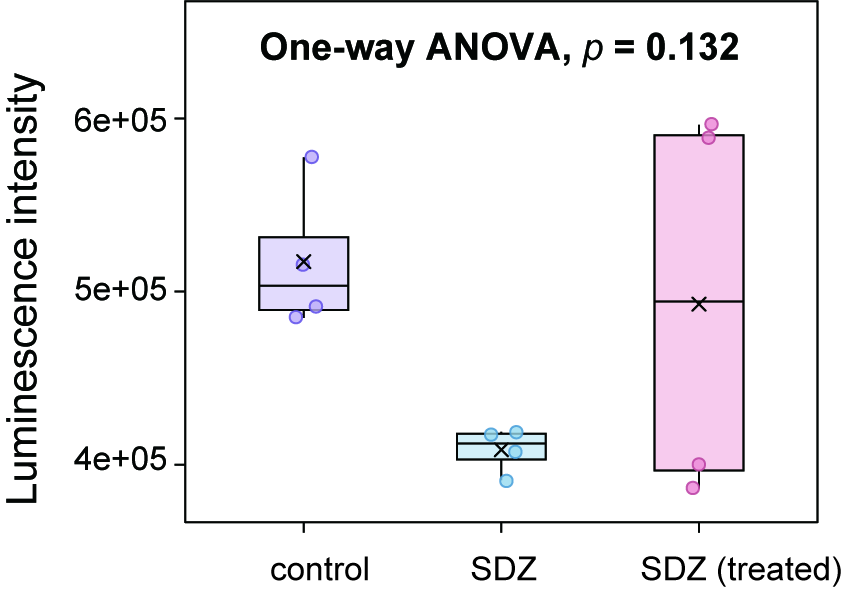


**Fig S15. Luminescence intensity of the Aliivibrio fischeri in different treatments.**


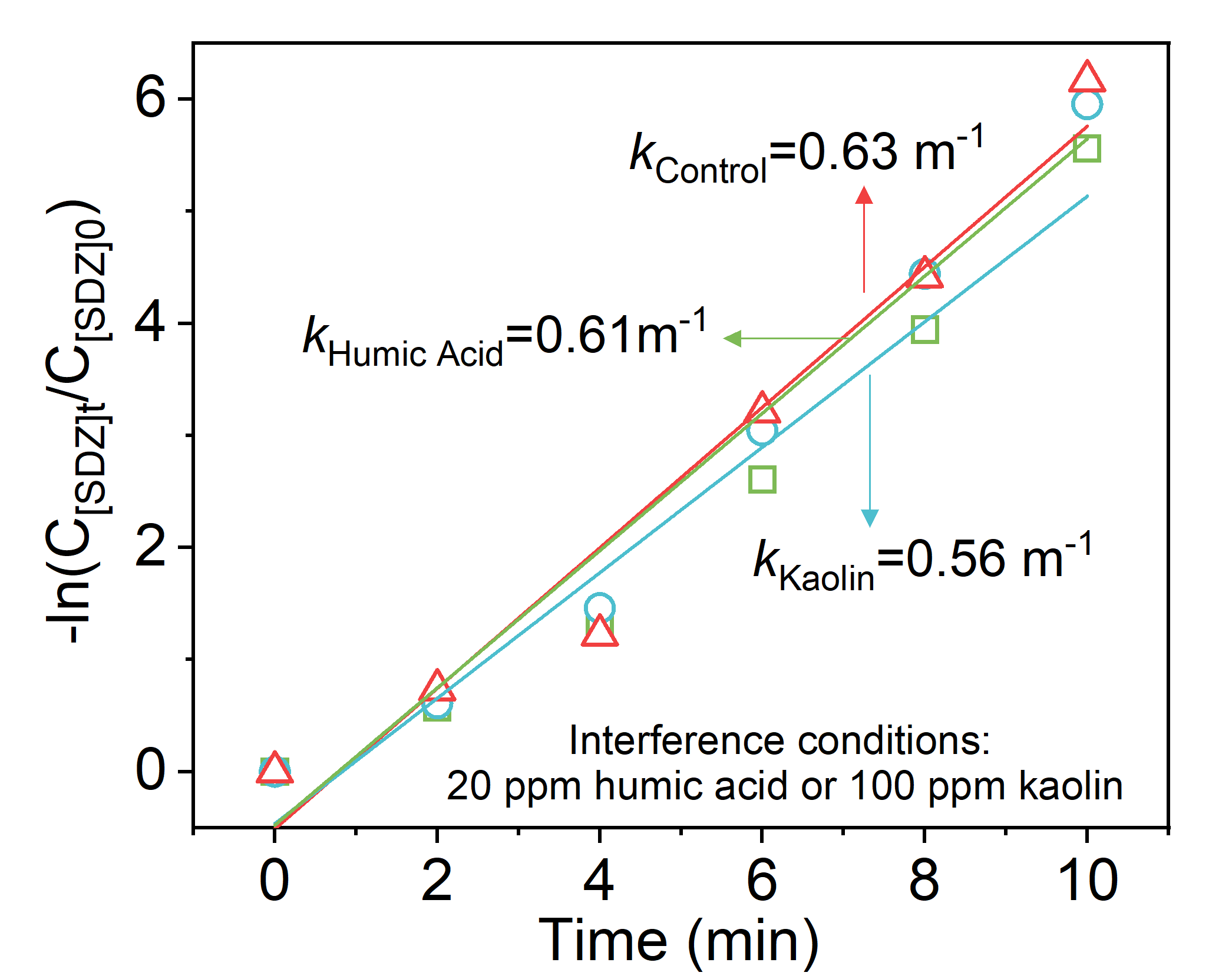


**Fig. S16. Effect of humic acid and kaolin on SDZ degradation kinetics in the CEC–VUV system.** Sulfadiazine degradation followed apparent pseudo-first-order kinetics under control conditions and in the presence of 20 ppm humic acid or 100 ppm kaolin. The apparent rate constant decreased slightly from 0.63 min^-1^ in the control system to 0.61 min⁻¹ with humic acid and 0.56 min^-1^ with kaolin, indicating that the CEC–VUV system retained strong degradation capability under deliberately challenging natural organic matter and turbidity interference conditions.


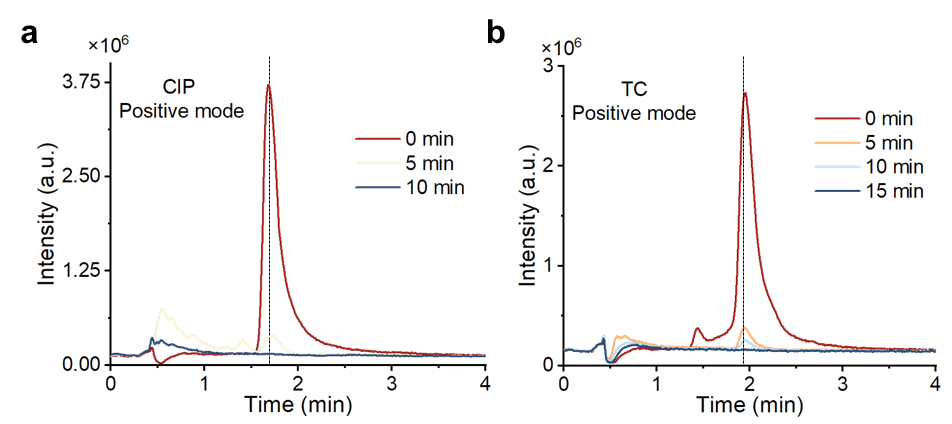


**Fig. S17.** **Degradation of (a) CIP and (b) TC detected by HPLC-MS.**

**Table S2**. Ultrasound-mediated pollutant degradation performance.

| **Source of energy** | **Catalyst** | **Pollutants** | | **Time and degree of** **degradation (min, %)** | | **Concentration**  **(ppm)** | **Ref.** |
| --- | --- | --- | --- | --- | --- | --- | --- |
| VUV (10W) & Ultrasonication  (40 kHz, 110 W) | PTFE | | SDZ, CIP, TC | | 10,100 | 20 | This Work |
| Ultrasonication  (40 kHz, 120 W) | 2.5PTFE/ZSM-5 | | MO | | 10, 98.5 | 10 | ^7^ |
| Ultrasonication  (40 kHz, 200 W) | FEP/Cu | | MO | | 120,100 | 5 | ^8^ |
| Ultrasonication  (40 kHz,500 W) | FEP | | Phenol | | 90,100 | 94.1 (1mM) | ^9^ |
| Ultrasonication  (40 kHz, 120 W) | FEP | | MO | | 120, ~100 | 5 | ^10^ |
| Ultrasonication  (40 kHz, 100 W) | Bi_12_O_17_Cl_2_ | | Acetaminophen (ACE) | | 60, 80 | 10 | ^11^ |
| Ultrasonication  (40 kHz, 152 W) | BIO | | Sulfamethazine (SMZ) | | 30, 98 | 20 | ^12^ |
| Ultrasonication  (40 kHz, 120 W) | Fe_3_O_4_ @SiO_2_ | | MO | | 180, 97 | 5 | ^13^ |
| Visible light  (300 W Xe lamp) & Ultrasonication  (45 kHz, 200 W) | BT@TiO_2_ | RhB | | | 75, 99.5 | 30 | ^14^ |
| Ultrasonication  (20 kHz, 500 W) | BaTiO_3−x_ | RhB | | | 450, 86 | 10 | ^15^ |
| Visible light  (300 W Xe lamp)  & Ultrasonication  (40 kHz, 100 W) | BTO/CPSS | RhB | | | 30, 90.6 | 5 | ^16^ |
| Ultrasonication  (40 kHz, 300 W) | CZO-2 | Phenol | | | 180, 98.7 | 10 | ^17^ |

**Table S3**. Pollutant degradation performance compare with other methods.

| **Catalytic method** | **Target pollutant** | **Pollutant concentration (ppm)** | **Degradation rate (min^-1^)** | **Reference** |
| --- | --- | --- | --- | --- |
| Contact-electro-catalysis + VUV irradiation  (This Work) | Sulfadiazine | 20 | 0.48 | This Work |
| Photocatalysis | Penicillin G | 33.44  (0.1 mM) | 0.0258 | ^18^ |
| Photocatalysis | Levofloxacin Hydrochloride | 10 | 0.0394 | ^19^ |
| Electrocatalysis  (50.0 mM Na_2_SO_4_  as supporting electrolyte ) | Tetracycline | 10 | 0.362 | ^20^ |
| Electrocatalysis  (50.0 mM Na_2_SO_4_  as supporting electrolyte ) | Ciprofloxacin | 30 | 0.06 | ^21^ |
| Electrocatalysis  (No supporting electrolyte) | Trichloroacetic acid | 0.12 | No provided  (120 min 99%) | ^22^ |
| Pre-oxidant (0.2 mM PAA) + Fenton-like catalyst | Bisphenol A | 33.44  (0.1 mM) | 0.188 | ^23^ |


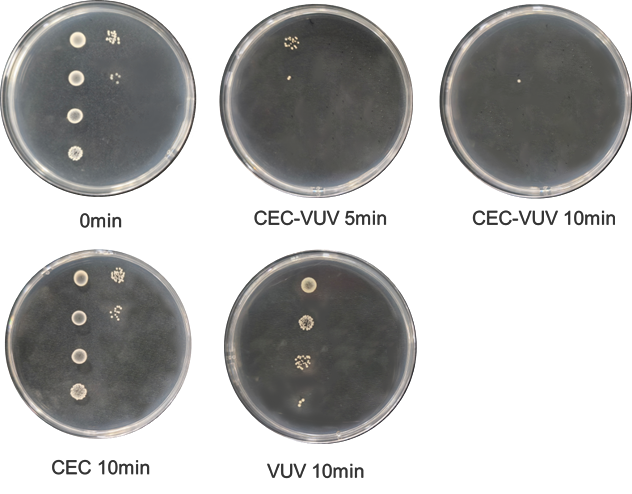


**Fig. S18. The inactivation efficiency of different systems against *S. aureus* was assessed using the plate counting method.**

Bacterial suspensions before and after treatment were serially diluted with sterile water (from 10 to 10^-6^), and 5 μL of each dilution was dropped onto LB agar plates. The plates were incubated at 37 °C for 16 hours. The results showed that S. aureus was completely inactivated (100%) within 10 minutes under the combined CEC-VUV treatment. The CEC system alone lacked significant bactericidal efficacy, whereas the VUV system displayed moderate antibacterial activity but did not achieve complete disinfection.


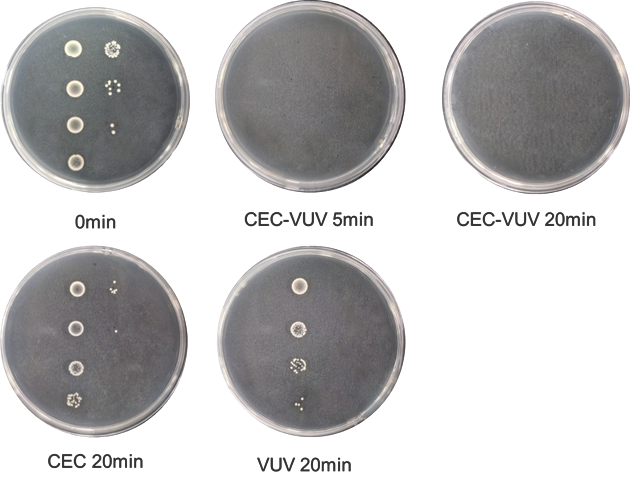


**Fig. S19. The inactivation efficiency of different systems against *E. coli* was evaluated using the plate counting method.**


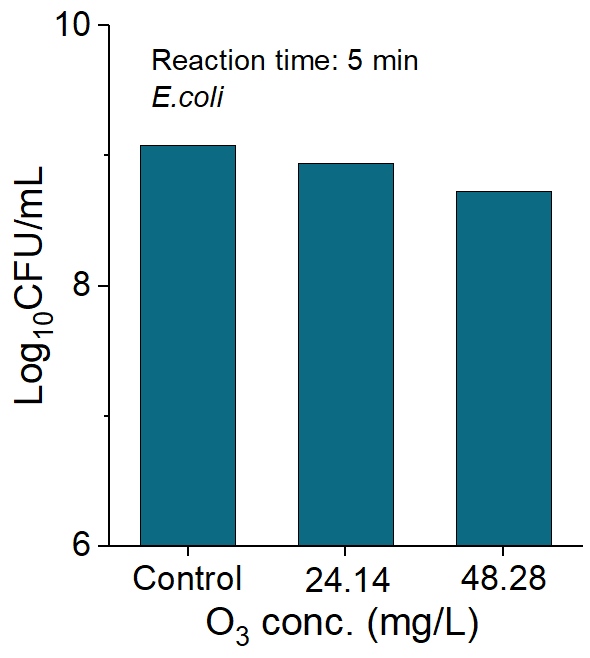


**Fig. S20. Inactivation rate of *E. coli* at different ozone concentrations.**


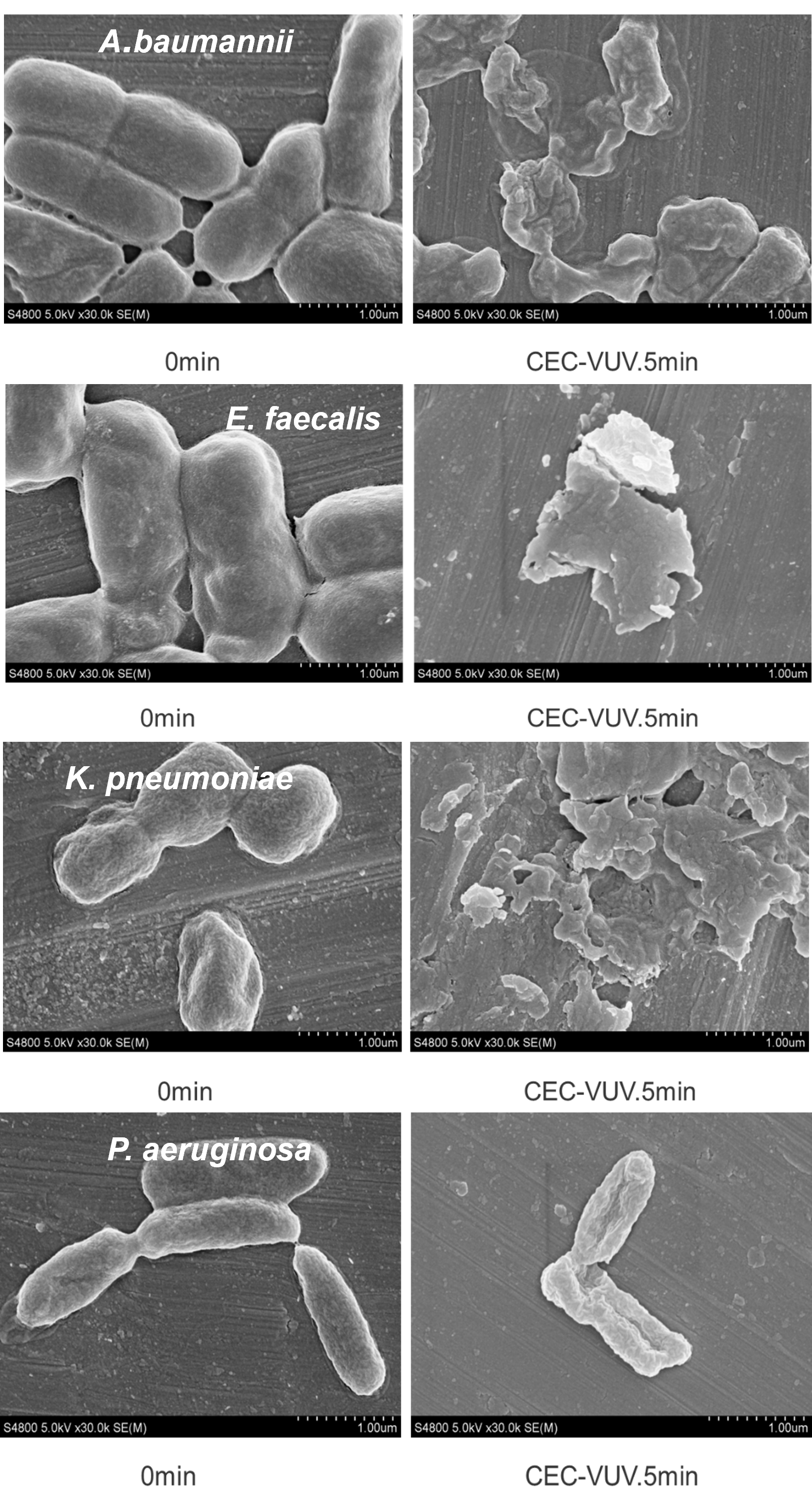


**Fig. S21. SEM images of *A. baumannii*, *E. faecalis*, *K. pneumoniae*, and *P. aeruginosa* before and after CEC-VUV treatment revealed significant structural damage to all four pathogenic bacteria following treatment.**

The cells exhibited marked disruption of their morphology, with some bacteria even disintegrating into cellular debris.


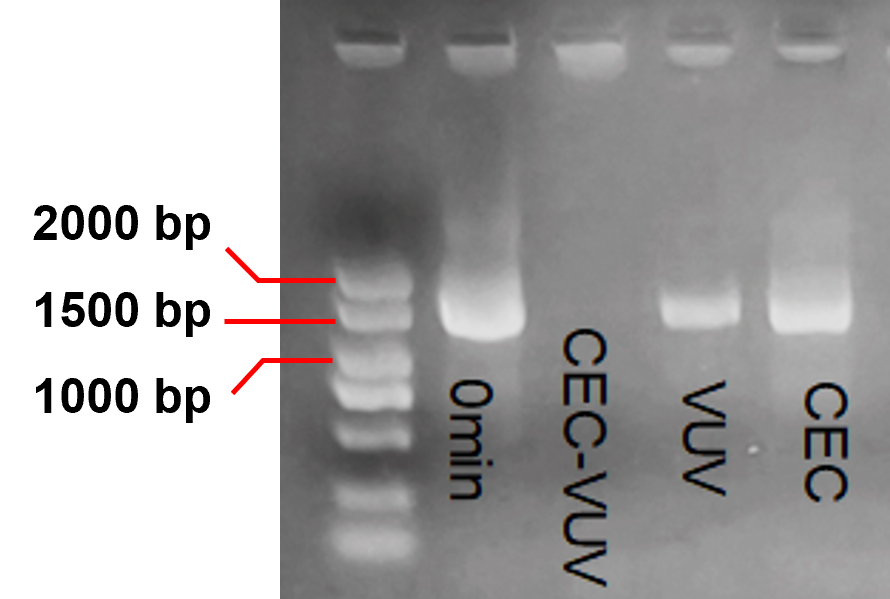


**Fig. S22. Gel electrophoresis images of full length 16S rRNA gene products amplified by PCR for Xinglin Bay water samples before and after treatments.**

**Table S4.** Primer sequences used for quantitative real-time polymerase chain reaction (qPCR).

| **Primer** | **Sequences (5'-3')** |
| --- | --- |
| 16S_27F  16S_1492R  16S_1132F  16S_1108R  sul1_F  sul1_R  tetA_F  tetA_R  intI1_F  intI1_R | AGAGTTTGATCMTGGCTCAG  TACGGYTACCTTGTTACGACTT  ATGGYTGTCGTCAGCTCGTG  GGGTTGCGCTCGTTGC  GCCGATGAGATCAGACGTATTG  CGCATAGCGCTGGGTTTC  CTCACCAGCCTGACCTCGAT  CACGTTGTTATAGAAGCCGCATAG  GCCTTGATGTTACCCGAGAG  GATCGGTCGAATGCGTGT |


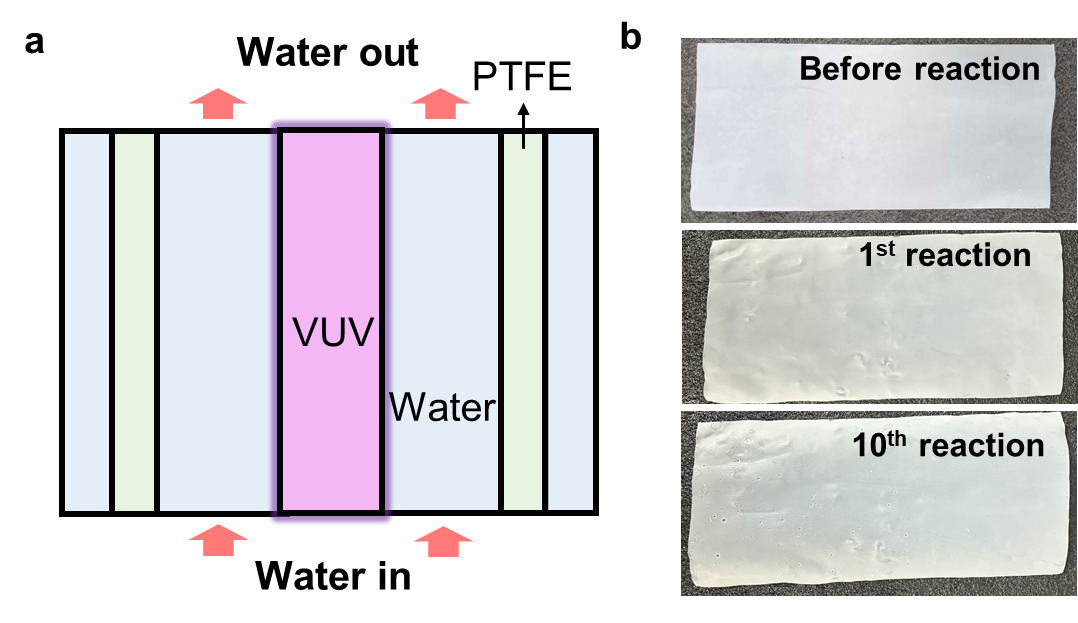


**Fig. S23.** **Effect of CEC-VUV system on PTFE membrane.** (a) PTFE membrane and VUV location in the reactor (side view). b. Comparative surface morphology of the PTFE membrane after different reaction counts. Each reaction lasted 10 min with an initial SDZ concentration of 20 ppm. High-frequency ultrasonication during operation induces wrinkling of the PTFE membrane and results in observable changes in surface morphology.


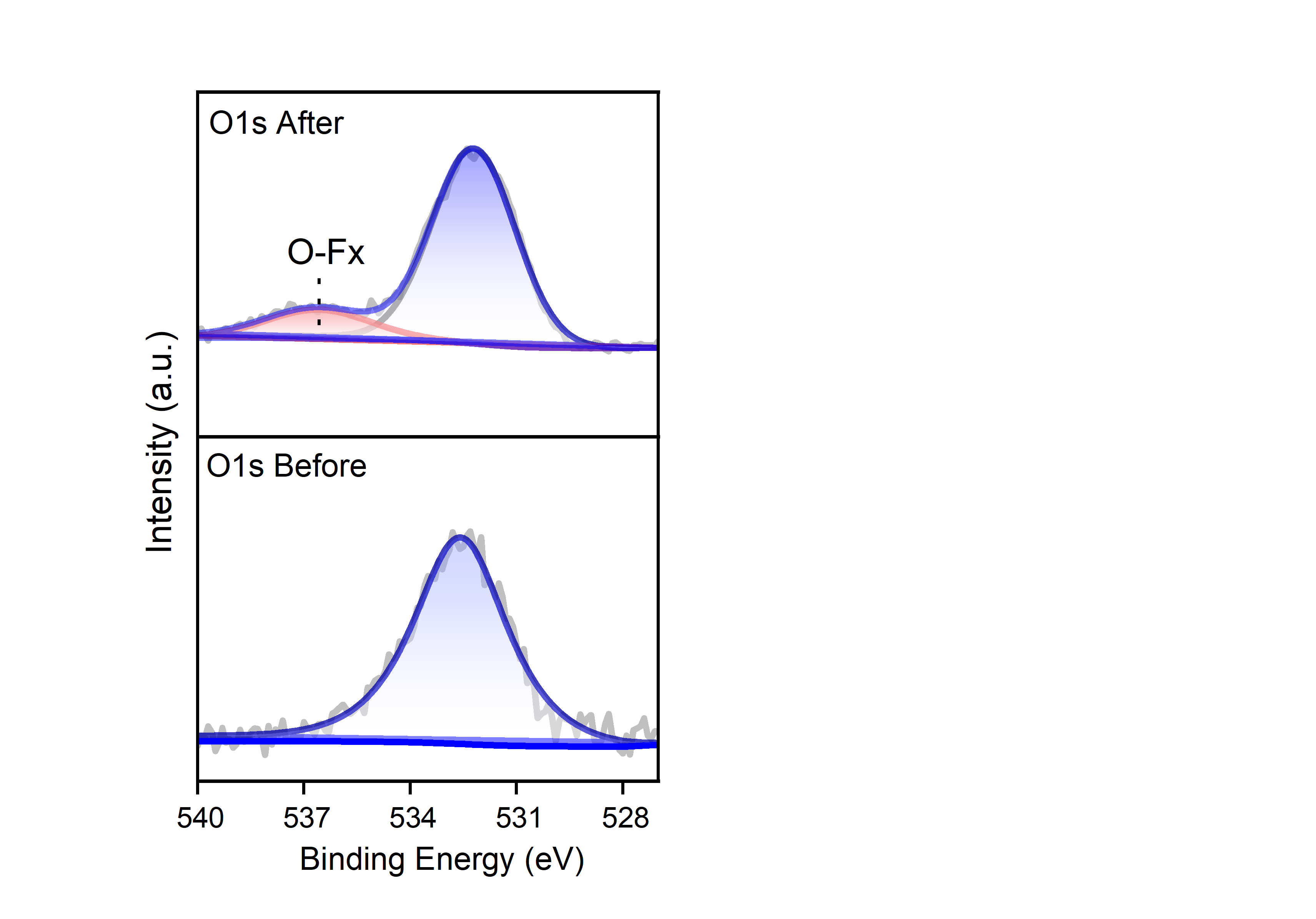


**Fig. S24. O1s signal of XPS of PTFE membrane before and after the reaction.**

The PTFE membrane was cleaned and dried after each reaction and tested after 10 cycles of antibiotic degradation experiments.


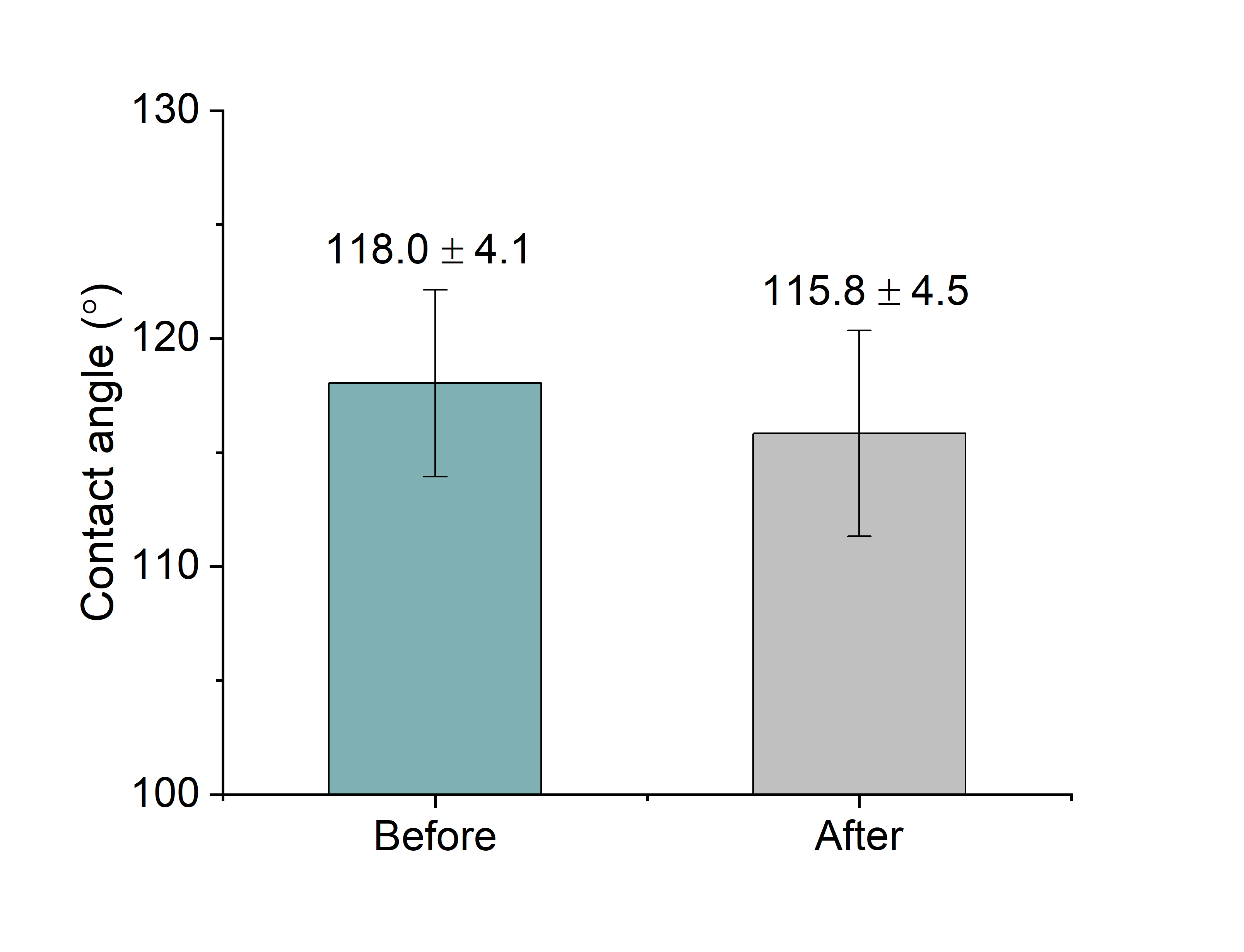


**Fig. S25. Static water contact angle measurements on the PTFE membrane before and after 10 reaction cycles.** With each cycle lasting 20 min, 6 distinct points were selected for water contact angle measurement before and after the reaction.


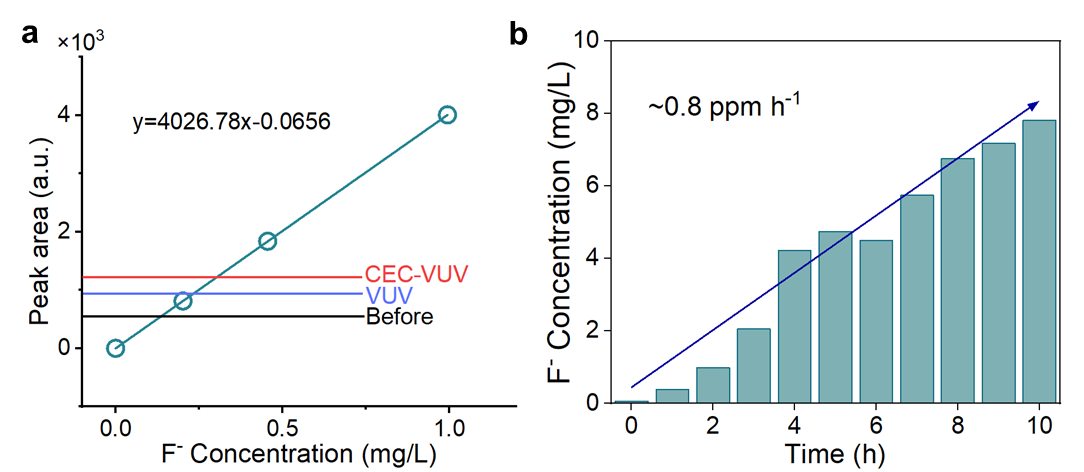


**Fig. S26. Ion chromatography detection of F^-^.** (a) In different systems (30 min) and quantitative standard curves . (b) Long-term defluorination over 10 h under closed conditions by reacting water with PTFE under CEC-VUV.

Before the reaction, shake the PTFE membrane and water thoroughly and extract 1 mL of solution. CEC-VUV and VUV were tested by extracting 1 mL of solution after half an hour of reaction under two conditions. Finally, the total F^-^ of the system was deducted from the F^-^ before the reaction.


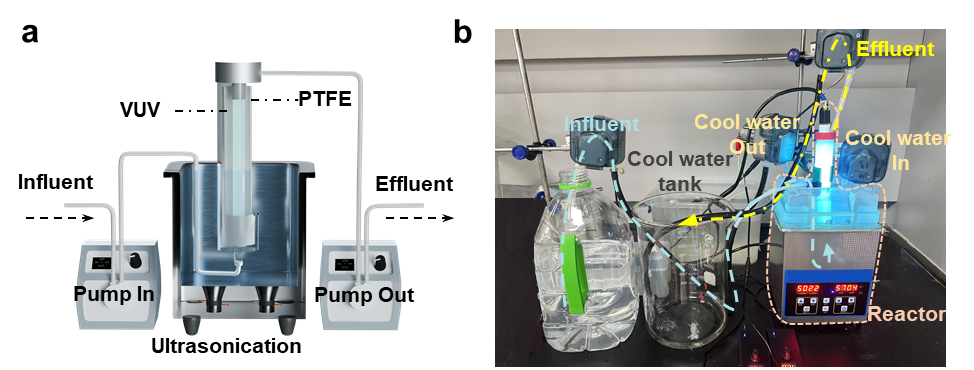


**Fig. S27. Schematic diagram of the continuous flow reaction system.** (a) Conceptual diagram and (b) actual diagram.


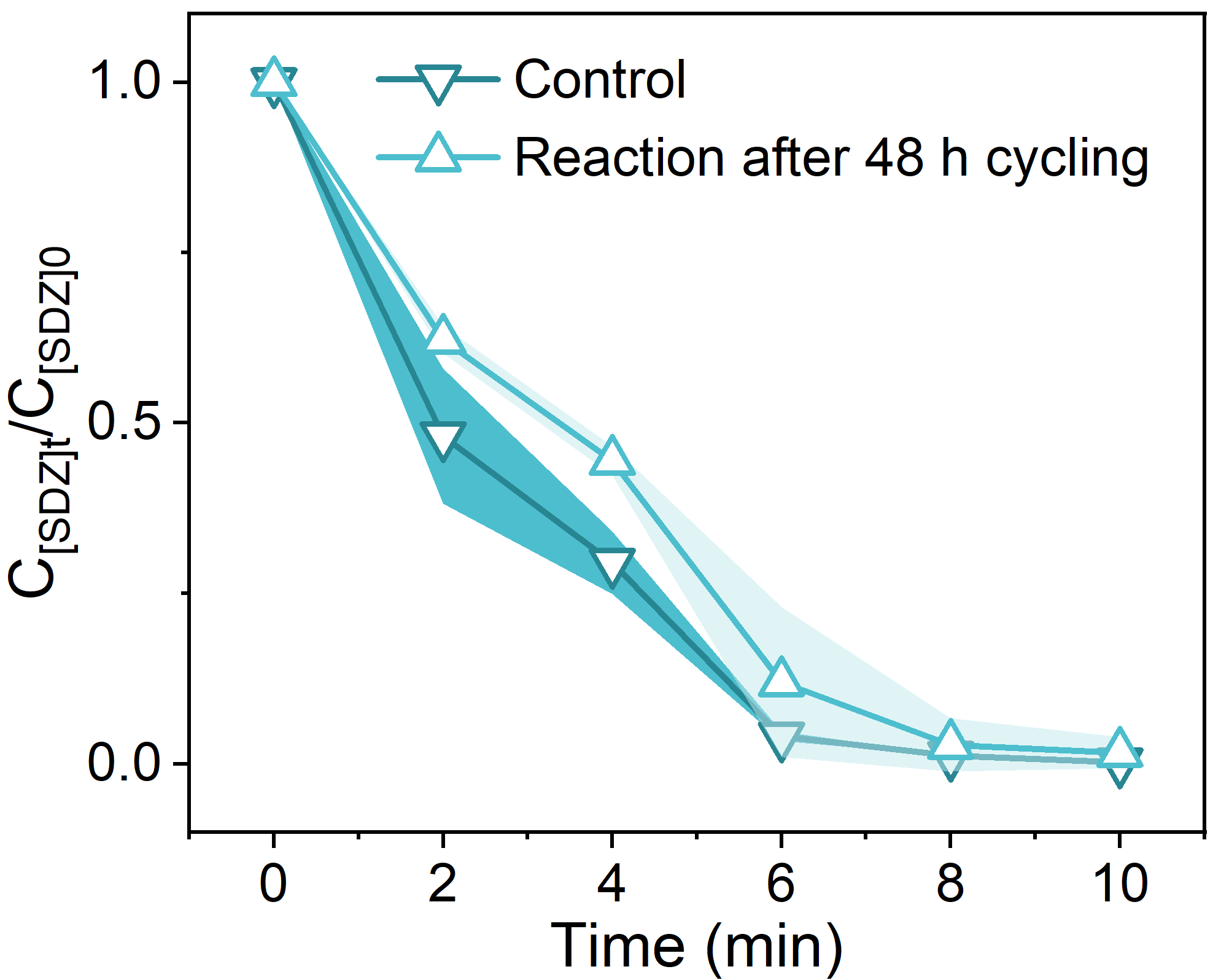


**Fig. S28. SDZ degradation performance of fresh PTFE and PTFE reused after 48 h recirculating operation in simulated complex water.** The simulated water contained 20 mg L^−1^ kaolin, 5 mg L^−1^ humic acid, 1 mM CaCl_2_, 2 mM NaHCO_3_, and 10 mM NaCl, which were used to represent suspended mineral particles, natural organic matter, inorganic scaling tendency, and background ionic strength, respectively. The solution was circulated through the CEC–VUV reactor at 500 mL min^−1^ for 48 h and then returned to the reservoir, corresponding to a cumulative treated volume of 1440 L. This experiment was designed to impose an accelerated fouling/scaling stress on the PTFE membrane surface rather than to simulate a simple single-pass filtration process.


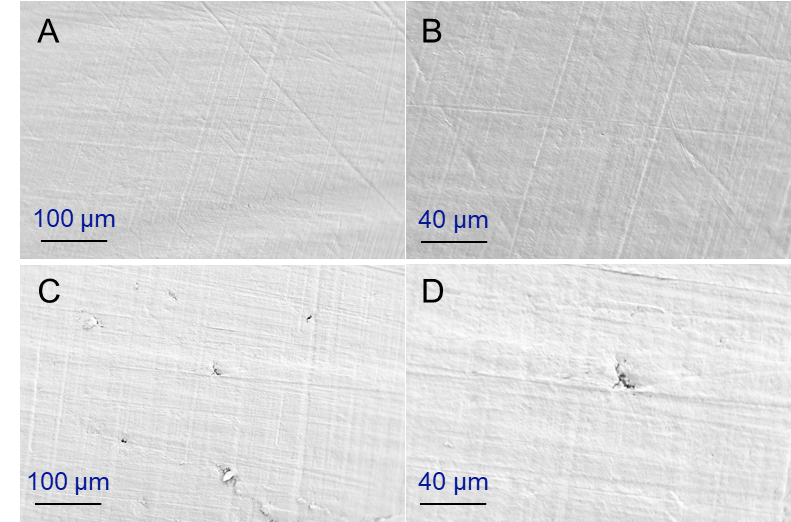


**Fig. S29. SEM images of the PTFE membrane before reaction and after 48h reaction.**

**Reference**

1 Delley, B. From molecules to solids with the DMol3 approach. *J. Chem. Phys.* **113**, 7756-7764 (2000). <https://doi.org/10.1063/1.1316015>

2 Perdew, J. P., Burke, K. & Ernzerhof, M. Generalized Gradient Approximation Made Simple. *Phys. Rev. Lett.* **77**, 3865-3868 (1996). <https://doi.org/10.1103/PhysRevLett.77.3865>

3 Delley, B. An all‐electron numerical method for solving the local density functional for polyatomic molecules. *J. Chem. Phys.* **92**, 508-517 (1990). <https://doi.org/10.1063/1.458452>

4 Dolg, M., Wedig, U., Stoll, H. & Preuss, H. Energy‐adjusted ab initio pseudopotentials for the first row transition elements. *J. Chem. Phys.* **86**, 866-872 (1987). <https://doi.org/10.1063/1.452288>

5 Bergner, A., Michael, D., Wolfgang, K., Hermann, S. & and Preuß, H. Ab initio energy-adjusted pseudopotentials for elements of groups 13–17. *Mol. Phys* **80**, 1431-1441 (1993). <https://doi.org/10.1080/00268979300103121>

6 Klamt, A. & Schüürmann, G. COSMO: a new approach to dielectric screening in solvents with explicit expressions for the screening energy and its gradient. *J. Chem. Soc. Perkin Trans. 2* 799-805 (1993). <https://doi.org/10.1039/P29930000799>

7 Li, W. *et al.* Boosting Reactive Oxygen Species Generation via Contact-Electro-Catalysis with Fe^III^-Initiated Self-cycled Fenton System. *Angew. Chem. Int. Ed.* **64**, e202413246 (2025). <https://doi.org/10.1002/anie.202413246>

8 Dong, X. *et al.* Regulating Contact-Electro-Catalysis Using Polymer/Metal Janus Composite Catalysts. *J. Am. Chem. Soc.* (2024). <https://doi.org/10.1021/jacs.4c07446>

9 Liu, J. *et al.* Nonaqueous Contact-Electro-Chemistry via Triboelectric Charge. *J. Am. Chem. Soc.* **146**, 31574-31584 (2024). <https://doi.org/10.1021/jacs.4c09318>

10 Wang, Z. *et al.* Contact-electro-catalysis for the degradation of organic pollutants using pristine dielectric powders. *Nat. Commun.* **13**, 130 (2022). <https://doi.org/10.1038/s41467-021-27789-1>

11 Xu, J. *et al.* Highly Efficient Fe(III) -initiated Self-cycled Fenton System in Piezo-catalytic Process for Organic Pollutants Degradation. *Angew. Chem. Int. Ed.* **62**, e202307018 (2023). <https://doi.org/10.1002/anie.202307018>

12 Wu, Y. *et al.* Triggering Dual Two-electron Pathway for H_2_O_2_ Generation by Multiple [Bi-O]n Interlayers in Ultrathin Bi_12_O_17_Cl_2_ towards Efficient Piezo-self-Fenton Catalysis. *Angew. Chem. Int. Ed.* **63**, e202316410 (2024). <https://doi.org/10.1002/anie.202316410>

13 Chen, Z., Lu, Y., Liu, X., Li, J. & Liu, Q. Novel magnetic catalysts for organic pollutant degradation via contact electro-catalysis. *Nano Energy* **108**, 108198 (2023). <https://doi.org/10.1016/j.nanoen.2023.108198>

14 Liu, Q. *et al.* Piezo-photoelectronic coupling effect of BaTiO_3_@TiO_2_ nanowires for highly concentrated dye degradation. *Nano Energy* **92**, 106702 (2022). <https://doi.org/10.1016/j.nanoen.2021.106702>

15 Ji, M., Kim, J. K., Ryu, C.-H. & Lee, Y.-I. Synthesis of self-modified black BaTiO_3-x_ nanoparticles and effect of oxygen vacancy for the expansion of piezocatalytic application. *Nano Energy* **95**, 106993 (2022). <https://doi.org/10.1016/j.nanoen.2022.106993>

16 Chen, Z. *et al.* Facile synthesis of advanced BaTiO3/CuPbSbS3 heterostructure photocatalyst with enhanced piezo-photocatalytic degradation performance. *Nano Energy* **124**, 109463 (2024). <https://doi.org/10.1016/j.nanoen.2024.109463>

17 Ran, M. *et al.* Dynamic defects boost in-situ H_2_O_2_ piezocatalysis for water cleanup. *Proc. Natl. Acad. Sci. U.S.A.* **121**, e2317435121 (2024). <https://doi.org/10.1073/pnas.2317435121>

18 Li, J. *et al.* Cu<sup>2+</sup> coordination-induced in situ photo-to-heat on catalytic sites to hydrolyze &#x3b2;-lactam antibiotics pollutants in waters. *Proc. Natl. Acad. Sci. U. S. A.* **120**, e2302761120 (2023). <https://doi.org/doi:10.1073/pnas.2302761120>

19 Bai, C. W. *et al.* Dynamic in-situ reconstruction of active site circulators for photo-Fenton-like reactions. *Nat. Commun.* **16**, 3019 (2025). <https://doi.org/10.1038/s41467-025-58392-3>

20 Lu, S., Li, X., Cheng, Y., Zhou, J. & Zhang, G. In situ electrogenerated Cu(III) triggers hydroxyl radical production on the Cu-Sb-SnO_2_ electrode for highly efficient water decontamination. *Proc. Natl. Acad. Sci. U. S. A.* **120**, e2306835120 (2023). <https://doi.org/doi:10.1073/pnas.2306835120>

21 Xie, L. *et al.* The strong metal-support interactions induced electrocatalytic three-electron oxygen reduction to hydroxyl radicals for water treatment. *Proc. Natl. Acad. Sci. U. S. A.* **120**, e2307989120 (2023). <https://doi.org/10.1073/pnas.2307989120>

22 Xu, Y. *et al.* Electrochemical hydrogenation of oxidized contaminants for water purification without supporting electrolyte. *Nat. Water* **1**, 95-103 (2023). <https://doi.org/10.1038/s44221-022-00002-3>

23 Liu, T. *et al.* Water decontamination via nonradical process by nanoconfined Fenton-like catalysts. *Nat. Commun.* **14** (2023). <https://doi.org/10.1038/s41467-023-38677-1>
